# Mesoporous Silica Nanoparticles Enhance the Anticancer Efficacy of Platinum(IV)-Phenolate Conjugates in Breast Cancer Cell Lines

**DOI:** 10.3390/nano12213767

**Published:** 2022-10-26

**Authors:** Ivana Predarska, Mohamad Saoud, Dijana Drača, Ibrahim Morgan, Teodora Komazec, Thomas Eichhorn, Ekatarina Mihajlović, Duško Dunđerović, Sanja Mijatović, Danijela Maksimović-Ivanić, Evamarie Hey-Hawkins, Goran N. Kaluđerović

**Affiliations:** 1 Faculty of Chemistry and Mineralogy, Institute of Inorganic Chemistry, Universität Leipzig, Johannisallee 29, 04103 Leipzig, Germany; 2 Department of Engineering and Natural Sciences, University of Applied Sciences Merseburg, Eberhard-Leibnitz-Str. 2, 06217 Merseburg, Germany; 3 Department of Bioorganic Chemistry, Leibniz Institute of Plant Biochemistry, Weinberg 3, 06120 Halle (Saale), Germany; 4 Institute for Biological Research “Siniša Stanković”, National Institute of Republic of Serbia, University of Belgrade, Bulevar despota Stefana 142, 11060 Belgrade, Serbia; 5 Institute of Pathology, School of Medicine, University of Belgrade, dr Subotića 1, 11000 Belgrade, Serbia

**Keywords:** platinum(IV) conjugates, cisplatin, phenolic acid, nanoparticles, drug delivery, breast cancer

## Abstract

The main reasons for the limited clinical efficacy of the platinum(II)-based agent cisplatin include drug resistance and significant side effects. Due to their better stability, as well as the possibility to introduce biologically active ligands in their axial positions constructing multifunctional prodrugs, creating platinum(IV) complexes is a tempting strategy for addressing these limitations. Another strategy for developing chemotherapeutics with lower toxicity relies on the ability of nanoparticles to accumulate in greater quantities in tumor tissues through passive targeting. To combine the two approaches, three platinum(IV) conjugates based on a cisplatin scaffold containing in the axial positions derivatives of caffeic and ferulic acid were prepared and loaded into SBA-15 to produce the corresponding mesoporous silica nanoparticles (MSNs). The free platinum(IV) conjugates demonstrated higher or comparable activity with respect to cisplatin against different human breast cancer cell lines, while upon immobilization, superior antiproliferative activity with markedly increased cytotoxicity (more than 1000-fold lower IC_50_ values) compared to cisplatin was observed. Mechanistic investigations with the most potent conjugate, cisplatin-diacetyl caffeate (**1**), and the corresponding MSNs (SBA-15|**1**) in a 4T1 mouse breast cancer cell line showed that these compounds induce apoptotic cell death causing strong caspase activation. In vivo, in BALB/c mice, **1** and SBA-15|**1** inhibited the tumor growth while decreasing the necrotic area and lowering the mitotic rate.

## 1. Introduction

Caffeic acid (CA) and ferulic acid (FA) belong to the most common phenolic acids, naturally occurring in fruits, grains, vegetables, spices, tea and coffee [1]. Apart from the key functions that these secondary plant metabolites exhibit on plant growth, development and defence, they are also found to have highly beneficial effects on humans. They are known for being potent natural antioxidants that are important for a variety of biological and pharmacological processes, such as cancer prevention, inflammation reduction, bacterial and viral infections prevention, blood clotting prevention, liver protection and many others [2,3,4,5,6,7,8]. The antioxidant properties of these phenolic acids are accredited to their chemical structure which includes one (ferulic acid) or two (caffeic acid) free phenolic hydroxyl groups and a double bond in the aliphatic chain enabling the formation of a phenoxy radical stabilized through resonance. In addition to the free radical scavenging via hydrogen atom donation [9], these phenolic acids are also found to modulate enzymatic activity, affect signal transduction as well as activate transcription factors and gene expression. As an example, alkyl esters of caffeic and ferulic acid are found to demonstrate significant inhibition on the activity of cyclooxygenase enzymes (COX-1 and COX-2) [10,11,12,13] responsible for the formation of key proinflammatory mediators. Additionally, COX-2 is overexpressed in a variety of tumors [14], making it a desirable target for the creation of anticancer medications. Therefore, in the past decade, these phenolic acids have attracted extensive attention as potential chemopreventive and chemotherapeutic agents. In that sense, multiple groups have pointed to the ability of caffeic acid and its derivatives to inhibit the growth of colon cancer [15,16,17,18], express anti-hepatocellular carcinoma activity [19], suppress the cancer cell proliferation in the human HT-1080 fibrosarcoma cell line [20] as well as human cervical cancer cells (HeLa and Me-180) [21] and reduce not only the growth of breast cancer, but also the ability of the breast cancer MCF-7 cells to migrate [22,23] which is a crucial factor for cancer metastasis. Ferulic acid as well has been found to have tumor-suppression potential in colon and breast cancers [4,24]. Additionally, both caffeic and ferulic acid have been found to increase the therapeutic efficacy of known anticancer drugs or lower the drug-related side effects. In this regard, the combined therapy involving caffeic acid and paclitaxel has been shown to have synergistic anticancer activity on H1299 lung cancer cells leading to enhanced apoptosis in vitro and in vivo [25]. Furthermore, caffeic acid elevates the antitumor effect of metformin in HTB-34 human metastatic cervical carcinoma cells [26] and increases the susceptibility of gastric cancer cells to cisplatin and doxorubicin [27]. Synergistic effects are also found applying cisplatin and caffeic acid in combination resulting in apoptotic mode of cell death in human cervical cancer cells [28]. A very interesting finding has been made by Sirota et al. who have discovered that in ovarian carcinoma cell lines the timing of caffeic acid treatment determines whether they become cisplatin-sensitive or -resistant [29]. Their findings demonstrate that preincubation with caffeic acid before treatment with cisplatin resulted in acquired resistance to cisplatin and decreased DNA binding, whereas simultaneous treatment with cisplatin and caffeic acid can increase cytotoxicity of cisplatin and platinum binding to nuclear DNA [29].

Based on all these findings and bearing in mind the different pharmacokinetic profiles of phenolic acids and cisplatin, including rising uncertainties whether these agents can reach the tumors within the required time frame in vivo, we have designed conjugates that combine the two agents into a single platinum(IV) prodrug. In this way, together with the molecules of the platinum component, the molecules of the respective phenolic acid can also be delivered into the tumor cells. Upon intracellular activation of these prodrugs via 2-electron reduction and protonation, the two moieties can be released and together carry out their biological functions in a synergistic manner [30]. Another reason which makes this approach attractive is that the coordinatively saturated platinum(IV) complexes are more stable than platinum(II) complexes, such as cisplatin, thus preventing its premature activation occurring by hydrolysis of the labile platinum chlorido ligand bonds, which is associated with many of its drawbacks such as the severe side effects, fast detoxification and innate or acquired resistance [31,32].

In an effort to create chemotherapeutics with lower toxicity, we have also used an SBA-15 mesoporous silica as nanocarrier for the platinum(IV) conjugates, aiming to take advantage of the accumulation of nanoparticles in tumor tissues through the passive enhanced permeability and retention (EPR) effect [33]. An additional advantage of this strategy is the possibility to further protect the drug from premature interaction with biomolecules on the way to cancer cells as well as provide tailorable drug release. The mesoporous silica SBA-15 material was chosen as a drug delivery vehicle because it is nontoxic to cells [34] and it has advantageous properties, including high pore volume with tuneable nanoscale pores as well as large surface area [35,36,37]. The properties of the inorganic mesoporous silica materials that make them interesting for biomedical applications, especially as nanoplatforms for drug delivery, have been highlighted in several reviews in the recent years [38,39,40,41,42,43,44]. The use of SBA-15 as a cisplatin carrier in earlier studies has been associated with increased cytotoxicity in leukemic cells [45], a twenty-fold greater antiproliferative effect in HT-29 colon cancer cells [46] and activation of pathways that change the phenotype of cancer cells (i.e., melanoma B16 tumor cells differentiate into senescent cells) [47]. Formerly reported SBA-15 nanoparticles loaded with platinum(IV) conjugates have also demonstrated very high antiproliferative activity against different breast cancer cell lines, while maintaining the structural integrity and allowing for relatively slow release of the active compounds [48].

**Figure 1:**
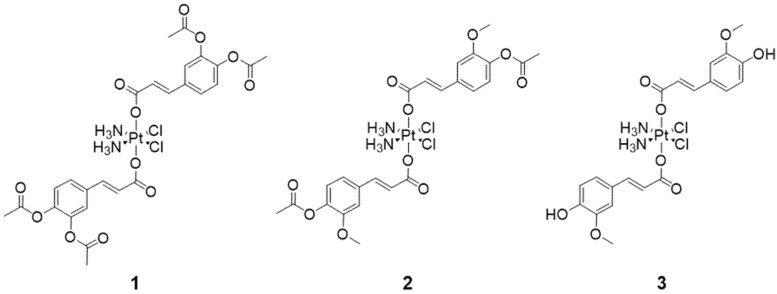
Platinum(IV)-phenolate conjugates: cisplatin-acetyl-protected caffeate (**1**), cisplatin-acetyl-protected ferulate (**2**) and cisplatin-ferulate (**3**).

Thus, we report here the preparation of three platinum(IV) derivatives of cisplatin bearing phenolates as axial ligands and their immobilization in SBA-15 nanoparticles. In these conjugates, both axial positions are occupied with acetyl-protected caffeate (**1**) or ferulate (**2**), as well as ferulate (**3**) (Figure 1). It should be noted that a cisplatin-ferulate conjugate has previously been reported [49]. However, in our view, the spectra included in the supplementary information of the corresponding paper do not confirm or support the structure of the desired product [49]. The prepared platinum(IV) complexes and their respective nanomaterials demonstrated remarkable antiproliferative activity in four different human breast cancer cell lines with different characteristics. In addition, synthesized conjugates and appropriate nanomaterials showed notable anticancer potential against a mouse breast cancer cell line as well. The activity of conjugate **1** and its corresponding MSNs SBA-15|**1**, which proved to be the most potent compounds in vitro, was further assessed in vivo, in a BALB/c mouse model with breast tumor induced by inoculation of 4T1 cells. **1** and SBA-15|**1** demonstrated potential to inhibit the tumor growth while decreasing the necrotic area and lowering the mitotic rate.

## 2. Materials and Methods

Standard Schlenk methods and anhydrous, degassed solvents were used to conduct all reactions in a nitrogen atmosphere. Acetone was first refluxed with KMnO_4_, then distilled from drierite (anhydrous CaSO_4_), pyridine was distilled from CaH_2_; both solvents were stored over activated 3 and 4 Å molecular sieves, respectively; MBraun Solvent Purification System (MB SPS-800) was used to obtain dry toluene, dichloromethane (DCM) and dimethylformamide (DMF), which were afterwards kept over activated 4 Å molecular sieves. Chemicals (cisplatin (Carbolution, St. Ingbert, Germany), caffeic acid (Fluorochem, Dublin, Ireland), ferulic acid, oxalyl chloride, tetraethyl orthosilicate (TEOS), pluronic 123 (P123) and acetic anhydride (Sigma-Aldrich Chemie GmbH, Steinheim, Germany)) were used as supplied. The synthesis of oxoplatin, *cis*,*trans*,*cis*-[PtCl_2_(OH)_2_(NH_3_)_2_], was performed according to an already reported procedure [50]. In short, cisplatin was oxidized with 30% (*w*/*w*) aqueous solution of hydrogen peroxide. Protection of the free phenolic hydroxy groups by acylation was done with acetic anhydride employing a literature-known procedure [51]. The acyl chloride derivative of acetyl-protected caffeic acid was prepared with oxalyl chloride and catalytic amounts of DMF in DCM [51], while for the preparation of the acyl chloride derivative of acetyl-protected ferulic acid, toluene was used as solvent.

A 400 MHz NMR spectrometer (BRUKER Avance III HD, Karlsruhe, Germany) was used for recording of ^1^H (400.13 MHz, tetramethylsilane (TMS) as an internal reference), ^13^C (100.63 MHz, internal reference TMS) and ^195^Pt (85.85 MHz, external reference Na_2_[PtCl_6_]), as well as two-dimensional ^1^H−^1^H COSY, ^1^H−^13^C HSQC, ^1^H−^13^C HMBC NMR spectra at 25 °C. The values of chemical shifts δ are given in parts per million (ppm). Electrospray ionization mass spectrometry was performed by using ESI-qTOF Impact II in positive mode (Bruker Daltonics GmbH, Bremen, Germany). Elemental analyses (C, H and N) were carried out with a microanalyser (VARIO EL, Heraeus Group, Hanau, Germany). VEGA3 (Tescan, Brno, Czech Republic) was employed for collection of scanning electron microscopy (SEM) images, as well as to conduct energy-dispersive X-ray spectroscopy (EDX) experiments (element detector EDAX Inc, Mahwah, NJ, USA). For nitrogen sorption measurements an Autosorb iQ/ASiQwin (Quantachrome Instruments, Anton Paar, QuantaTec Inc., Boynton Beach, FL, USA) was used. Small angle X-ray scattering (SAXS) measurements were performed on a D8 ADVANCE (Bruker, Karlsruhe, Germany) X-ray diffraction system. Inductively coupled plasma-optical emission spectrometry (ICP-OES) analysis was achieved on an Optima 7000 DV spectrometer (PerkinElmer, Waltham, MA, USA) using WinLab32 software.

### 2.1. Synthesis of Platinum(IV) Conjugates ***1*** and ***2***

Pyridine (0.48 mL, 6.0 mmol, 10 eq.) was added to an oxoplatin (0.2 g, 0.6 mmol, 1 eq.) suspension in acetone (12 mL). After 10 min stirring, acyl chloride derivative, dissolved in acetone (12 mL), of either acetyl-protected caffeic acid or acetyl-protected ferulic acid (0.85 g or 0.76 g, respectively, each 3.0 mmol, 5 eq.) was added. The reaction mixture was refluxed at 75 °C (48 h). The precipitate formed was filtrated and thoroughly washed (water: 30 mL × 3; ethanol: 10 mL × 3; diethyl ether: 10 mL × 3) to eliminate the pyridinium salt. After drying in air, the pure products were collected.

**1**, pale yellow powder. Yield: 0.32 g (64%).

**^1^H NMR (DMSO-*d*_6_, ppm):** *δ* = 7.63 (d, ^4^*J*_HH_ = 2 Hz, 2H, C*H*_aryl_), 7.60 (dd, ^3^*J*_HH_ = 8 Hz, ^4^*J*_HH_ = 2 Hz, 2H, C*H*_aryl_), 7.38 (d, ^3^*J*_HH_ = 16 Hz, 2H, C*H*_vinyl_), 7.30 (d, ^3^*J*_HH_ = 8 Hz, 2H, C*H*_aryl_), 6.80–6.50 (br, 6H, N*H*_3_), 6.59 (d, ^3^*J*_HH_ = 16 Hz, 2H, C*H*_vinyl_), 2.29 (s, 12H, OCOC*H*_3_).

**^13^C{^1^H} NMR (DMSO-*d*_6_, ppm):** *δ* = 174.0 (qC, *C*OO), 168.7 (qC, *C*OO), 143.4 (qC, *C*_aryl_), 142.9 (qC, *C*_aryl_), 139.9 (CH, *C*_vinyl_), 133.9 (qC, *C*_aryl_), 126.7 (CH, *C*_aryl_), 124.6 (CH, *C*_aryl_), 123.3 (CH, *C*_aryl_), 122.8 (CH, C_vinyl_), 20.9 (CH_3_, OCO*C*H_3_).

**^195^Pt{^1^H} NMR (DMSO-*d*_6_, ppm):** *δ* = 1213 (br s).

**HR-ESI-MS (positive mode, CH_3_OH):***m*/*z* [2M + H]^+^: calc. for C_52_H_57_Cl_4_N_4_O_24_Pt_2_: 1653.139, found: 1653.135; *m*/*z* [M + H]^+^: calc. for C_26_H_29_Cl_2_N_2_O_12_Pt: 827.073, found: 827.070.

**Elemental analysis:** Found: C, 37.95; H, 3.2; N 3.5. Calc. for C_26_H_28_Cl_2_N_2_O_12_Pt: C, 37.8; H, 3.4; N, 3.4%.

**2**, yellow powder. Yield: 0.27 g (59%).

**^1^H NMR (DMSO-*d*_6_, ppm):** *δ* = 7.45 (d, ^4^*J*_HH_ = 2 Hz, 2H, C*H*_aryl_), 7.39 (d, ^3^*J*_HH_ = 16 Hz, 2H, C*H*_vinyl_), 7.21 (dd, ^3^*J*_HH_ = 8 Hz, ^4^*J*_HH_ = 2 Hz, 2H, C*H*_aryl_), 7.11 (d, ^3^*J*_HH_ = 8 Hz, 2H, C*H*_aryl_), 6.80–6.50 (br, 6H, N*H*_3_), 6.65 (d, ^3^*J*_HH_ = 16 Hz, 2H, C*H*_vinyl_), 3.83 (s, 6H, OC*H*_3_), 2.27 (s, 6H, OCOC*H*_3_).

**^13^C{^1^H} NMR (DMSO-*d*_6_, ppm):** *δ* = 174.2 (qC, *C*OO), 168.9 (qC, *C*OO), 151.5 (qC, *C*_aryl_)148.4 (qC, *C*_aryl_), 140.9 (qC, *C*_vinyl_), 133.9 (qC, *C*_aryl_), 123.6 (CH, *C*_aryl_), 121.9 (CH, *C*_vinyl_), 121.3 (CH, *C*_aryl_), 111.7 (CH, *C*_aryl_), 59.3 (CH_3_, O*C*H_3_), 20.8 (CH_3_, OCO*C*H_3_).

**^195^Pt{^1^H} NMR (DMSO-*d*_6_, ppm):** *δ* = 1212 (br s).

**HR-ESI-MS (positive mode, CH_3_OH):***m*/*z* [2M + H]^+^: calc. for C_48_H_57_Cl_4_N_4_O_20_Pt_2_: 1541.159, found: 1541.141; *m*/*z* [M + H]^+^: calc. for C_24_H_29_Cl_2_N_2_O_10_Pt: 771.083, found: 771.086; *m*/*z* [M + Na]^+^: calc. for C_24_H_28_Cl_2_N_2_NaO_10_Pt: 793.065, found: 793.068.

**Elemental analysis:** Found: C, 37.6; H, 3.5; N, 3.9. Calc. for C_24_H_28_Cl_2_N_2_O_10_Pt: C, 37.4; H, 3.6; N, 3.65%.

### 2.2. Deprotection of ***2*** to Obtain Platinum(IV) Conjugate ***3***

In a mixture of DCM, methanol and acetone (20 mL of each solvent) conjugate **2** (0.3 g, 0.39 mmol) was dissolved. Conc. HCl was added dropwise (10 mL) to the colorless solution, and a color change to light yellow was noticeable after a few minutes. To track the progress of the reaction TLC (ethyl acetate:*n*-hexane = 3:1) was used. After 3 h stirring at r.t., the starting material was no longer present. The volume of the solvents was reduced to ca. 15 mL, subsequently water (50 mL) was added. The reaction mixture was filtrated and the isolated product was washed (water: 10 mL × 3; ethanol: 10 mL × 3; diethyl ether: 10 mL × 3) and dried in air.

**3**, dark yellow powder. Yield: 0.25 g (93%).

**^1^H NMR (DMSO-*d*_6_, ppm):** *δ* = 9.45 (s, 2H, O*H*), 7.30 (d, ^3^*J*_HH_ = 16 Hz, 2H, C*H*_vinyl_), 7.23 (d, ^4^*J*_HH_ = 2 Hz, 2H, C*H*_aryl_), 7.02 (dd, ^3^*J*_HH_ = 8 Hz, ^4^*J*_HH_ = 2 Hz, 2H, C*H*_aryl_), 6.80–6.50 (br, 6H, N*H*_3_), 6.78 (d, ^3^*J*_HH_ = 8 Hz, 2H, C*H*_aryl_), 6.42 (d, ^3^*J*_HH_ = 16 Hz, 2H, C*H*_vinyl_), 3.82 (s, 6H, OC*H*_3_).

**^13^C{^1^H} NMR (DMSO-*d*_6_, ppm):** *δ* = 174.9 (qC, *C*OO), 149.1 (qC, *C*_aryl_), 148.4 (qC, *C*_aryl_), 142.1 (CH, *C*_vinyl_), 126.5 (qC, *C*_vinyl_), 122.7 (CH, *C*_aryl_), 118.4 (CH, *C*_vinyl_), 115.9 (CH, *C*_aryl_), 111.1 (CH, *C*_aryl_), 56.1 (CH_3_, O*C*H_3_).

**^195^Pt{^1^H} NMR (DMSO-*d*_6_, ppm):** *δ* = 1216 (br s).

**HR-ESI-MS (positive mode, CH_3_OH):***m*/*z* [2M + H]^+^: calc. for C_40_H_49_Cl_4_N_4_O_16_Pt_2_: 1373.116, found: 1373.098; *m*/*z* [2M + Na]^+^: calc. for C_40_H_48_Cl_4_N_4_NaO_16_Pt_2_: 1395.098, found: 1395.068; *m*/*z* [2M + K]^+^: calc. for C_40_H_48_Cl_4_KN_4_O_16_Pt_2_: 1411.072, found: 1411.035; *m*/*z* [M + H]^+^: calc. for C_20_H_25_Cl_2_N_2_O_8_Pt: 687.062, found: 687.060.

**Elemental analysis:** Found: C, 35.1; H, 3.5; N, 4.2. Calc. for C_20_H_24_Cl_2_N_2_O_8_Pt: C, 35.0; H, 3.5; N, 4.1%.

### 2.3. Solubility and Stability of Conjugates ***1***–***3***

For determination of their water solubility, compounds **1**–**3** (5 mg, each) were suspended in distilled water (5 mL) and stirred for 12 h. After that, the undissolved solid was separated by filtration and the amount of dissolved conjugate was evaluated after evaporation of the solvent from the filtrate.

To deduce the stability of **1**–**3** in DMSO, time-resolved (0, 3, 6, 12, 24, 48 and 72 h) ^1^H NMR spectroscopy was applied.

### 2.4. Preparation of SBA-15

The SBA-15 silica mesoporous nanoparticles were prepared according to a reported procedure [52]. Briefly, to a solution of P123 (48.4 g) in a mixture of HCl (2 M, 1400 mL) and H_2_O (360 mL) at 35 °C, TEOS (102 g) was added dropwise. The reaction mixture was vigorously stirred at 35 °C (20 h), then at 80 °C (24 h). The solid material was isolated by filtration and dried at 90 °C (14 h). Finally, the material was heated to 500 °C (1 °C min^−1^) and kept for 24 h at this temperature for calcification.

Yield: 30.2 g; BET surface: 517 m^2^ g^−1^; wall thickness: 3.66 nm; pore diameter: 4.74 nm; pore volume: 0.72 cm^3^ g^−1^; lattice parameter: 8.4 nm; XRD (2*θ* in °, Miller indices): 1.0802 (100), 1.8032 (111), 2.0645 (200).

### 2.5. Preparation of SBA-15 Material Loaded with Platinum(IV) Conjugates, SBA-15|***1***–SBA-15|***3***

The literature procedure was followed for loading the conjugates **1**–**3** in the MSN mesopores [53,54]. Namely, to the activated SBA-15 nanoparticles (vacuum, 150 °C, 16 h), a suspension of **1**–**3** in toluene was added and the reaction mixture was agitated at 80 °C (48 h). Filtration was used to separate the solid material, which was then thoroughly washed using toluene and *n*-pentane. The MSNs loaded with drug (**1**–**3**) were obtained and dried at r.t.. Physical parameters of prepared MSNs are given in Table 1.

SBA-15|**1**: 165 mg of **1**, 200 mg of SBA-15; yield: 340 mg.

SBA-15|**2**: 231 mg of **2**, 300 mg of SBA-15; yield: 498 mg.

SBA-15|**3**: 103 mg of **3**, 150 mg of SBA-15; yield: 244 mg.

### 2.6. Drug Release Studies

ICP-OES was used to track the release of the conjugates **1**–**3** from the MSNs in phosphate-buffered saline (PBS) depending on the amount of platinum in the liquid phase. Experiments were performed by suspending the drug-loaded MSN material (2 mg) in PBS (1 mL) and sampling at defined times (0.08, 0.5, 1, 3, 6, 12, 24 and 72 h). Quantification of platinum in the PBS was done by ICP-OES upon separation of the solid material by centrifugation. External platinum standard solutions, obtained from an ICP platinum standard reference solution (Pt 1000 μg mL^−1^, Specpure^®^, Alfa Aesar GmbH & Co KG, Germany), were used for the calibration of the ICP-OES device. The measurement was carried out on the ^194^Pt isotope on a previously chosen emission line of platinum of 265.9 nm. Using different mathematical models (zero/first order, Korsmeyer-Peppas and Higuchi), the drug release kinetics were analyzed.

In addition, SBA-15|**1** and SBA-15|**2** (2 mg each) were each suspended in PBS (1 mL). The PBS was exchanged with fresh PBS (1 mL) every hour for 10 h consecutively. Again, the platinum present in the liquid phase was quantified using ICP-OES.

### 2.7. In Vitro Studies

ATCC (Manassas, VA, USA) provided all four human breast cancer cell lines. The mouse breast cancer cell line 4T1 (derived from the mammary gland tissue of a mouse BALB/c strain), was a kind gift from Prof. Nebojša Arsenijević from the Faculty of Medical Sciences, University of Kragujevac, Serbia. PBS, fetal calf serum (FCS) and basal cell culture media Roswell Park Memorial Institute (RPMI) were purchased from both Sigma-Aldrich (St. Louis, MO, USA) and Capricorn Scientific GmbH (Ebsdorfergrund, Germany). Trypsin/ethylenediaminetetraacetic acid (EDTA) and L-glutamine were bought from Capricorn Scientific GmbH (Ebsdorfergrund, Germany). Paraformaldehyde (PFA) was obtained from Serva (Heidelberg, Germany). DMSO, carboxyfluorescein diacetate succinimidyl ester (CFSE), propidium iodide (PI) and crystal violet (CV) were purchased from Sigma-Aldrich (St. Louis, MO, USA). The penicillin/streptomycin solution was obtained from Biological Industries (Cromwell, CT, USA). Acridine orange (AO) was obtained from Labo-Moderna (Paris, France). PAN Biotech GmbH (Aidenbach, Germany) supplied us with Endopan 3 with supplementing kits. ApoStat was purchased from R&D Systems (Minneapolis, MN, USA) while Annexin V-FITC (AnnV) was from Biolegend (San Diego, CA, USA). 4-Amino-5-methylamino-2′,7′-difluorofluorescein diacetate (DAF-FM diacetate) and dihydrorhodamine 123 (DHR) were bought from Thermo Fisher Scientific (Waltham, MA, USA). The multi-well plates, culture flasks and other cell culture plastics were obtained from TPP (Trasadingen, Switzerland) and Greiner Bio-One GmbH (Frickenhausen, Germany).

### 2.8. Cell Culture

In this work, the following human breast cancer cell lines were used: triple-negative breast adenocarcinoma (MDA-MB-468 and HCC1937), ER- and HER2-positive breast cancer (BT-474 and MCF-7) [55] as well as one mouse-derived breast cancer cell line (4T1). RPMI-1640 medium containing 2 mM L-glutamine, heat-inactivated FCS (10%), and penicillin/streptomycin (1%) was used to sustain all cell lines. Prior to use or further subculturing, the cell lines were cultured in T-75 flasks at 37 °C in a humidified environment with 5% CO_2_. Before cell passaging and seeding, the adherent cells were washed with PBS and detached by using trypsin/EDTA (0.05% in PBS) [54].

### 2.9. Cell Viability Studies

Using the above-mentioned cell growth media, cells were seeded in 96-well plates in a density of 6000 cells per 100 μL per well for the in vitro investigations in human breast cancer cell lines. Following seeding, cells were given a 24-h period to adhere before being treated with platinum(IV) conjugates **1**–**3** and MSNs loaded with **1**–**3**, namely SBA-15|**1**, SBA-15|**2** and SBA-15|**3**. Stock solutions of platinum(IV) conjugates **1**–**3**, phenolic acids (ligand precursors) and cisplatin were prepared in DMSO. Serial dilution was afterwards done in standard growth media to reach the following concentrations: 10, 5, 1, 0.5, 0.1, 0.01 and 0.001 μM for the conjugates, 100, 50, 25, 12.5, 6.25, 3.125 and 1.6 μM for the phenolic acids, and 300, 100, 30, 10, 3, 1, and 0.1 μM for cisplatin. In a repeated experiment, HCC1937, MCF-7 and BT-474 cells were treated with various concentrations of the conjugates **1**–**3**, namely 50, 25, 12.5, 6.25, 3.125, 1.6, 0.8 and 0.4 μM. Stock suspensions of SBA-15 and drug-loaded SBA-15 were prepared in PBS and serially diluted with standard growth media to achieve final concentrations of 50, 10, 5, 1, 0.5, 0.1, 0.01 and 0.001 μg mL^−1^ for drug-loaded SBA-15, and 100, 50, 25, 12.5, 6.25, 3.125 and 1.6 μg mL^−1^ for SBA-15 alone. Each 96-well plate contained a positive control using digitonin (100 µM). Three independent experiments were performed in quadruplicate.

For the in vitro studies in the mouse-derived breast cancer cell line, cells were seeded in a density of 2 × 10^3^ cells per well (96-well plates) using the previously indicated cell growth media. Following seeding, cells were given a 24 h adhesion period before being treated with conjugate **1** and SBA-15|**1**. Using a DMSO stock solution of **1**, prepared at a concentration of 10 mM and retained at −20 °C for 3 days, a serial dilution was made in standard growth media to achieve concentrations of 25, 12.5, 6.3, 3.12, 1.56, 0.78 and 0.39 µM. A stock suspension of SBA-15|**1** was prepared in PBS and serially diluted in standard growth media until final concentrations of 50, 25, 12.5, 6.25, 3.12, 1.56 and 0.78 μg mL^−1^ were reached. A DMSO solution of cisplatin at a concentration of 10 mM was prepared freshly before usage, and serial dilutions were made in culture medium to reach concentrations of 100, 50, 25, 12.5, 6.25, 3.12 and 1.56 µM. To determine the outcome of induced autophagy, cells were treated with an IC_50_ dose of conjugate **1** and SBA-15|**1** and concomitantly with 3-methyladenine (3-MA, 1 mM).

Cell viability was evaluated in each case after a 72-h incubation period. Colorimetric MTT- (3-(4,5-dimethylthiazol-2-yl)-2,5-diphenyltetrazolium bromide) and CV (crystal violet)-based cell viability assays were used to examine the compounds’ potential cytotoxicity. The MTT test was carried out in the following way: cells were rinsed with PBS before being incubated at 37 °C in a humid environment with 5% CO_2_ with an MTT standard solution (0.5 mg mL^−1^ MTT in culture medium). After one hour, the MTT solution was discarded and DMSO was used to dissolve the formazan that had formed. The absorbance of this formazan, along with the reference/background signal, could be measured at wavelengths of 540 nm and 670 nm. Measurements were taken utilizing SpectraMax M5 multi-well plate reader (MolecularDevices, San Jose, CA, USA). For the CV assay, cells were fixed with 4% PFA for 20 min at room temperature after being washed once with PBS. The PFA solution was then removed and the cells were left to dry for 10 min. Afterwards, cells were stained with 0.2% crystal violet solution for 20 min followed by removal of the staining solution, washing of the cells with water and letting them dry overnight at room temperature. Finally, upon addition of acetic acid (33% in aqua bidest.) to the stained cells, the absorbance was measured at reference wavelengths of 540 nm and 670 nm as described earlier [56]. A four-parametric logistic function was used to obtain the mean value of the cell viability, which is expressed as a percentage in comparison to untreated cells [57]. SigmaPlot 14.0 and Microsoft Excel 2013 were employed for data analyses and IC_50_ and MC_50_ calculation.

### 2.10. Flow Cytometry

Six-well plates were seeded with 4T1 cells (5 × 10^4^ per well). After overnight adherence, cells were exposed to an IC_50_ dose of **1** and an MC_50_ dose of SBA-15|**1** and analyzed by flow cytometry. There were several staining procedures used: (1) CFSE for observing the influence on cellular proliferation, (2) AnnV/PI for identifying apoptotic cells, (3) ApoStat for caspase activity detection, (4) AO for autophagy detection, (5) DHR for reactive oxygen/nitrogen species (ROS/RNS) measurement, (6) DAF-FM for intracellular nitric oxide (NO) measurement, and (7) PI for cell cycle analysis. Results were acquired by CyFlow^®^ Space Partec using the PartecFloMax^®^ software v1.0.0 (Partec GmbH, Münster, Germany), with an exception for the results of cell cycle analysis which were obtained using BD FACSAria III and BD FACSDiva software v8.3 (BD Biosciences, Franklin Lakes, NJ, USA). Three separate replicates of the experiment were run. Depending on the particular staining agent, channels FL1 (green emission), FL2 (orange emission), and/or FL3 (dark red emission) were used for fluorescence detection.

For AnnV/PI, ApoStat, and AO staining, after 72 h treatment with the investigated agents, the cells were trypsinized and rinsed with PBS. Afterwards, cells staining according to the manufacturer’s protocols was performed, with AnnV/PI in AnnV-binding buffer (15 min, room temperature), ApoStat in PBS 5% FBS (30 min, 37 °C) and AO 10 µM in PBS (15 min, 37 °C). Finally, upon washing, cells were resuspended in PBS (or in AnnV-binding buffer for AnnV/PI), and analyzed. For CFSE staining, pre-staining with PBS solution of CFSE (1 μM; 10 min, 37 °C) followed by washing and seeding of the cells was done, after which they were exposed to experimental agents over a period of 72 h. Before analysis, cells were once again washed, trypsinized and suspended in PBS. Similarly, pre-staining with 1 μM DHR for 20 min at 37 °C was first done also for DHR staining. For DAF-FM staining, the cells were first subjected to the experimental agents for 72 h, washed with PBS, and then stained with 5 μM DAF-FM diacetate in phenol red-free RPMI-1640 for 1 h at 37 °C. Thereafter, the dye was discarded and the cells were washed with PBS. Additional incubation of the cells in fresh RPMI-1640 not containing phenol red and serum, was done for 15 min to finish the reaction of de-esterification. Afterwards, cells were trypsinized, resuspended in PBS and analyzed. For analysis of cell distribution across cell cycle phases, after the treatments, 4T1 cells were fixed with 70% ethanol overnight at 4 °C, and then stained with PI (20 μg mL^−1^) in the presence of RNase (0.1 mg mL^−1^) during 45 min at 37 °C.

### 2.11. In Vivo Studies

Inbred female BALB/c mice (6–8 weeks old) from the Institute for Biological Research “Siniša Stanković”—National Institute of Republic of Serbia (IBISS) were used for this study. Animals were kept in standard laboratory conditions, free of non-specific pathogens, with unlimited access to food and water. The handling of animals and the study protocol adhered to national regulations established by the Law on Animal Welfare of the Republic of Serbia (Official Gazette of the Republic of Serbia No. 41/2009) and European Ethical Normative (Directive 2010/63/EU) on the protection of animals used for experimental and other scientific purposes. The national licensing committee at the Department of Animal Welfare, Veterinary Directorate, Ministry of Agriculture, Forestry and Water Management of Republic of Serbia granted approval for the experimental protocols (permission No. 323-07-07906/2022-05).

### 2.12. Induction of Tumors and In Vivo Treatment

BALB/c mice were orthotopically inoculated with 4T1 cells (2 × 10^4^ cells in 50 µL PBS) in the fat pad region of the fourth mammary gland region. On the fifth day following cell implantation when tumors were palpable, random assignment of the animals into different treatment groups was made. The administration regime consisted of i.p. application of the appropriate agent three times a week. Cisplatin was applied in a dose of 2 mg kg^−1^ in 2% DMF/PBS, conjugate **1** at 5 and 10 mg kg^−1^ in 2% DMSO/PBS, while SBA-15|**1** was applied in PBS at a dose of 17.5 and 35 mg kg^−1^ for 25 days. Control mice were receiving 2% DMSO/PBS and 2% DMF/PBS as vehicle. Tumor growth was monitored every second day and on the 29th day after cell inoculation, mice were sacrificed. Tumors were extracted and measured in three dimensions. The tumor volume was calculated based on the following equation:

V = 0.52 × *a* × *b*^2^

where “*a*” is the longest and “*b*” the shortest diameter. On the day of sacrifice, urine was collected from the animals and different biochemical parameters were analyzed with Multistix 10 SG (Bayer, Leverkusen, Germany).

### 2.13. Histopathological Examination

Harvested tissues of sacrificed animals (tumor, liver and kidney) were macroscopically examined, measured and sectioned to 3 mm thick slices through the largest tissue plane. Tissues were processed in automatic tissue processor (Milestone SRL LOGOS ONE, Sorisole, BG–Italy). Embedding in paraffin blocks was done on embedding console (SAKURA Tissue-Tek TEC 5, Sakura Finetek, CA, USA). Sections 4 µm thick were cut from the tissue using microtome (LEICA RM 2245, (Leica Biosystems, Nussloch, Germany), and the slices were mounted on glass slides. The slides were then stained with hematoxilin eosin stain (H/E) in automated slide stainer MYREVA SS-30H (Especialidades Médicas MYR, S.L., Tarragona, Spain). After H/E staining, all glass slides were examined and microscopically analyzed under an Olympus BX43 microscope (OLYMPUS EUROPA HOLDING GMBH, Hamburg, Germany). Additionally, all slides were digitalized with a Leica Aperio AT2 slide scanner (Leica Biosystems, Nussloch GmbH, Germany) for analysis and documentation purposes. Morphometric analysis was done with a Leica Aperio ImageScope (version 12.4.6, Leica Biosystems, Nussloch GmbH, Germany).

### 2.14. Statistical Analysis

Differences between treatments were assessed using analysis of variance (ANOVA) accompanied with a Student-Newman-Keuls test. Results of in vivo experiments were evaluated by a Mann-Whitney test. Differences in histopathological changes between treatments were analyzed by a Oneway ANOVA test followed by a Tukey test. Statistical significance was considered if *p*-value was 0.05 or less. Statistical analyses were done using the program Statistica 10.

## 3. Results and Discussion

### 3.1. Synthesis and Characterization of Platinum(IV) Conjugates ***1***–***3***

In this study, three cisplatin derivatives with acetyl-protected caffeate, acetyl-protected ferulate or free ferulate in the axial positions (complexes **1**, **2** and **3**, respectively, Figure 1) were prepared. Complexes **1** and **2** were synthesized by reacting oxoplatin (afforded by cisplatin oxidation with hydrogen peroxide) [50] with an excess of the corresponding acyl chloride derivative of the acetyl-protected phenolic acid in the presence of pyridine as base. Acetyl-protected phenolic acids were used for the synthesis in order to avoid side reactions involving the hydroxyl groups present in their structure. Additionally, as reported by other authors, the reduction potential of the phenolic acids is directly linked to the existence as well as the number of hydroxyl groups [58,59]. Therefore, using caffeic and ferulic acid with free hydroxyl groups could induce reduction of the platinum(IV) in oxoplatin making the formation of the desired products impossible [60,61]. After complexes **1** and **2** were successfully obtained in yields of 64% and 59%, respectively, deprotection of **2** was done with conc. HCl to afford the cisplatin-ferulate conjugate **3**. The three obtained platinum(IV) conjugates were characterized by ^1^H, ^13^C and ^195^Pt NMR spectroscopy and mass spectrometry (ESI, Figures S1–S18) and their purity was further confirmed through elemental analysis. Deprotection of **1** was also attempted, employing different procedures and reagents, from conc. HCl to ones which have been reported to be mild and highly selective for deprotection of aromatic acetates such as guanidine·HCl [62], dibutyltin oxide [63] and ammonium acetate [64]. However, the desired cisplatin-caffeate conjugate could not be obtained; instead, degradation of the complex resulting in very complex ^1^H NMR spectra was observed in each deprotecting approach. Finally, we also attempted the deprotection using a commercially available enzyme Amano lipase A from *Aspergillus niger* (Sigma-Aldrich Chemie GmbH, Steinheim, Germany) which has been reported to cause full *O*-deacetylation of different acetylated biomolecules under mild conditions [65]. Closely following the reaction by time-resolved ^1^H NMR spectroscopy (ESI, Figure S19), we noticed that as soon as the deacetylation of **1** begins, chemical shifts corresponding to free caffeate appear. This means that after deprotection, the free hydroxyl groups might reduce the platinum(IV) to platinum(II), leading to separation of the caffeate ligand from the cisplatin scaffold. This confirms the capacity of phenolic acids to induce metal reduction and the higher reduction potential of caffeic acid, which bears two hydroxyl groups in *ortho* position to each other, by comparison with ferulic acid, which has only one free hydroxyl group. As the deprotection of the acetyl protecting groups can occur under acidic conditions, it could be anticipated that the acidic tumor microenvironment would facilitate such a reaction in vivo. The free hydroxyl groups would then ease the reduction of the platinum(IV) to the cytotoxic platinum(II) and release the axial ligands, making both species available to act upon their respective targets.

The platinum(IV) conjugates are particularly poorly soluble in water (**1**–**3**: 0.09 g, 0.04 g and 0.56 g, respectively, in 1 L H_2_O) owing to the presence of lipophilic moieties. However, in polar aprotic solvents like DMF and DMSO, they are highly soluble.

As confirmed by time-resolved ^1^H NMR spectroscopy, the conjugates **1**–**3** are stable in DMSO solution over 72 h. During this time, no ligand exchange or complex degradation was observed (Figures S20–S22).

### 3.2. Synthesis and Characterization of the Mesoporous Silica Materials

**Figure 2:**
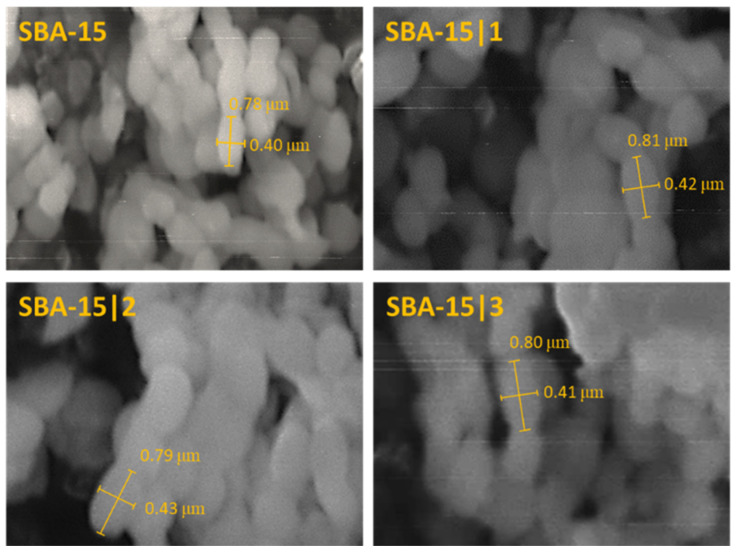
SEM images of MSNs: SBA-15, SBA-15|**1,** SBA-15|**2** and SBA-15|**3**.

For the preparation of SBA-15 nanoparticles, a synthesis described in the literature, with TEOS as a silica source and P123 serving as a structure-directing agent, was employed [52]. Calcination was performed to remove the organic template, resulting in rod-shaped particles with consistent morphology as demonstrated by SEM imaging (Figure 2). Narrow size distribution of the particles ranging from 200–400 × 600–800 nm was observed.

**Figure 3:**
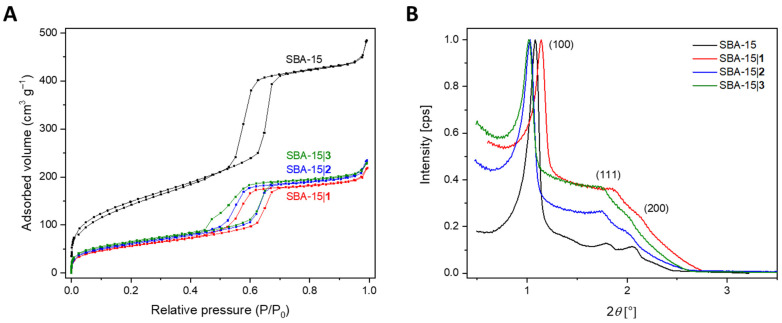
(**A**) N_2_ adsorption-desorption isotherms and (**B**) SAXS patterns of MSNs: SBA-15, SBA-15|**1**–SBA-15|**3**.

Nitrogen physisorption experiments that resulted in a type IV isotherm with a hysteresis loop showing capillary condensation characteristic for highly organized mesoporous structures (Figure 3A) [66] were used to confirm the material’s mesoporous nature. Carrying out these analyses also upon loading of the SBA-15 material with the platinum(IV) complexes **1**–**3** which afforded the three materials SBA-15|**1**, SBA-15|**2** and SBA-15|**3**, it was shown that the conjugates have not significantly influenced the SBA-15 structure, so the shape, morphology as well as the mesoporous nature of the materials are retained.

As expected, after loading of the cisplatin derivatives, the high specific surface area, *S*_BET_ (calculated based on the Brunauer–Emmett–Teller (BET) model), and pore volume, *V*_p_, which were corroborated for the SBA-15 material (517 m^2^ g^−1^ and 0.72 cm^3^ g^−1^, respectively), were reduced to 245−292 m^2^ g^−1^ for the specific surface area and 0.34–0.37 cm^3^ g^−1^ for the pore volume. Additionally, there was a change of the pore diameter *D*_p_ from 4.74 nm as determined for the pure SBA-15 to 3.68−4.75 nm for the loaded MSNs. On the contrary, the wall thickness *W*_p_, calculated by deducting the pore diameter from the lattice parameter *a*, increased upon immobilization of conjugates **2** and **3**, (3.66 nm 4.26 and 5.03 nm (SBA-15, SBA-15|**2** and SBA-15|**3**, respectively). The lattice parameter itself was attained by SAXS, and specifies the repetition rate of the hexagonal pores of the MSNs. Thus, the acquired data showing similar values for *a* for all pure and loaded MSNs are consistent with what is expected. Finally, as shown in Figure 3B, the SAXS patterns of pure SBA-15 and SBA-15|**1**–SBA-15|**3** exhibit diffraction peaks (see Table 1) typical for hexagonally ordered MSNs, highlighting the particles’ unaltered structure once more. All corresponding values for *a* and 2*θ* are presented in Table 1.

The amount of immobilized platinum(IV) complexes **1**–**3** in the MSNs was calculated based on the amount of platinum detected with EDX analysis. Successful loading of all three compounds **1**–**3** with load contents of 7.51, 9.23 and 10.33 wt% Pt, respectively, was confirmed. The corresponding encapsulation efficiency was calculated to be 70.4%, 83.8% and 89.2%, for SBA-15|**1**–SBA-15|**3**, respectively (see ESI, Figure S23 and Tables S1 and S2).

Despite the successful production of the mesoporous material with the applied method, which resulted in MSNs with optimal drug loading, it is worth mentioning that significant advances have been made in the engineering of MSNs [67], including some greener approaches with less undesirable environmental impact [68], which should be considered in the future.

### 3.3. Release of Platinum(IV) Conjugates from MSNs

**Figure 4:**
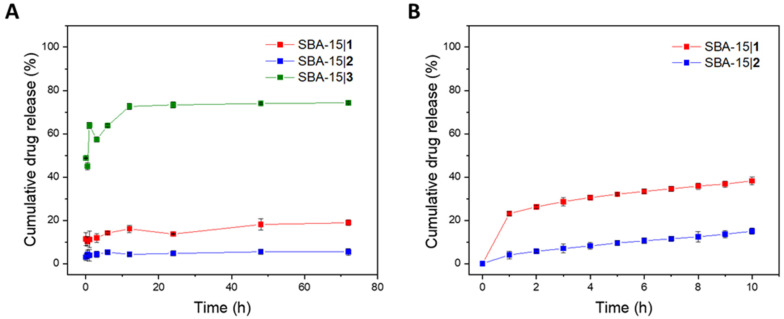
(**A**) Cumulative drug release (%) of the respective platinum(IV) conjugate from SBA-15|**1**, SBA-15|**2** and SBA-15|**3** over 72 h; (**B**) Cumulative drug release (%) of the respective platinum(IV) conjugate from SBA-15|**1** and SBA-15|**2** with exchange of the PBS solution every hour for 10 h.

The in vitro release profiles of the platinum(IV) complexes **1**–**3** encapsulated in silica nanoparticles were determined in PBS (pH = 7.4) by assessing the platinum content in solution (ICP-OES) at predetermined time points (0.08, 0.5, 1, 3, 6, 12, 24, 48 and 72 h). As presented in Figure 4A, SBA-15|**1** and SBA-15|**2** exhibited very low release of only 18 and 5%, respectively, over 72 h. On the contrary, SBA-15|**3** released over 70% of its cargo over the same period of time. As these results are consistent with the solubility profiles of the three conjugates in water, it is probable that in this case drug release is driven by solubility of the drugs in the surrounding media. In the first hours, saturation of the solution is achieved and, therefore, prolonged contact with the fluid does not lead to increase the amount of platinum(IV) conjugate released. By exchanging the medium each hour in the time interval of up to 10 h, saturation of PBS with the drugs from nanomaterials SBA-15|**1** and SBA-15|**2** is avoided. As initially observed, the release is low, but continuous (Figure 4B). The obtained results indicate that the drug-loaded nanoparticles’ structural integrity is maintained under the simulated physiological setting, preventing immediate leakage of the platinum(IV) complexes. In vivo, this could, in addition to the favorable EPR effect of MSNs, be very beneficial as it could imply minimal drug release during circulation, thus lowering the possibility of off-target interactions and side effects, and higher accumulation in the tumor, where the slow release could ensure prolonged drug exposure.

Mathematical models *viz*. zero/first order, Korsmeyer–Peppas and Higuchi (see ESI, Figures S24–S26) were employed for a study of the kinetics of the drug release [69]. Data was quantitatively correlated with these models and the most suitable model was determined based on the degree of correlation R^2^ (see ESI, Table S3). The results obtained suggest that although the release of the cisplatin derivatives from the corresponding MSNs does not follow perfectly the principles of any of the investigated release kinetics (R^2^ < 0.9), diffusion-controlled drug release kinetics (Higuchi for SBA-15|**1** and Korsmeyer–Peppas for SBA-15|**2** and SBA-15|**3**) are the most fitting [70]. The value *n* obtained from the Korsmeyer–Peppas model (in all cases n < 0.45) further ascertained Fickian diffusion as prime release mechanism [71].

### 3.4. Antiproliferative Activity

The antiproliferative activity of the free platinum(IV) complexes **1**, **2** and **3** and the corresponding MSNs (SBA-15, SBA-15|**1**–SBA-15|**3**) was first determined against four human breast tumor cell lines, involving two triple-negative cell lines, namely MDA-MB-468 (human breast adenocarcinoma with COX-2 expression) and HCC1937 (human breast carcinoma with BRCA1 mutation), and the two ER- and PR-positive cell lines MCF-7 (with COX-1 expression) and BT-474 (both invasive ductal breast carcinoma). After 72 h of treatment with the examined compounds and MSNs, the cell viability was assessed using the two distinct assays, CV and MTT, and their activity was compared with that of clinically used cisplatin, as well as the phenolic acids used as ligands. IC_50_ and MC_50_ values are presented in Table 2. As expected, the ligands alone were found to be inactive (>100 μM) against all treated tumor cell lines. A similar observation was made for the SBA-15 material (>100 μg mL^−1^). On the contrary, the platinum(IV) conjugates **1–3** demonstrated antitumor activity in the range similar to (against MDA-MB-468 and HCC1937 cell lines) or higher (against MCF-7 and BT-474 cell lines) than cisplatin. Certain discrepancies were observed between the results acquired by MTT and CV assays against the MCF-7 cell line upon treatment with the platinum(IV) conjugates and against BT-474, HCC1937 and MDA-MB-468 cell lines upon treatment with cisplatin, implying that in these cases the mechanism through which cytotoxicity is achieved might be involving cell metabolism pathways [72,73,74,75].

Immobilization of the platinum(IV) complexes **1**–**3** in MSNs led to a significant improvement of the antiproliferative activity of all three conjugates against all cell lines involved in the study. For comparative evaluation with the activity of cisplatin and the free platinum(IV) complexes, recalculation of the IC_50_ values for the drug-loaded MSNs (SBA-15|**1**, SBA-15|**2** and SBA-15|**3**) was made based on the obtained MC_50_ values considering the platinum content obtained by EDX, as well as the release of the drugs obtained by ICP-OES (detailed explanation in ESI). Submicromolar IC_50_ values which were at least two times and in some cases several thousand times lower than those for cisplatin were obtained. All drug-loaded MSNs showed the highest activity against the BT-474 cell line responsible for the development of invasive ductal breast cancer and known to be resistant to the conventional treatment with tamoxifen [76,77]. Among the three drug-loaded MSNs, SBA-15|**1** was found to have the highest potency against this cell line with an IC_50_ value of only 0.1 nM.

Considering these very promising results, further investigations were necessary in an appropriate in vivo breast cancer model; thus, an orthotopic model of breast cancer by inoculation of 4T1 cells in BALB/c mice was selected. Therefore, the antiproliferative activity of the most active conjugate **1** and appropriate nanomaterial SBA-15|**1** was first assessed in vitro in this triple-negative mouse cell line. After 72 h of incubation, the viability of the cells was determined by measurement of total cell culture respiration or number of adherent cells using MTT and CV assays. The results summarized in Table 2 reveal that conjugate **1** strongly affected the cell viability of 4T1 cells similarly as cisplatin alone. Higher activity exceeding the one of cisplatin and **1** was observed upon drug-loading into SBA-15 particles, which is consistent with the results seen in the human breast cancer cell lines. Cell viability under **1**–**3**, SBA-15|**1**–SBA-15|**3** and cisplatin exposure is presented in the ESI, Figures S27–S31. 

### 3.5. Molecular Mechanism of Action

**Figure 5:**
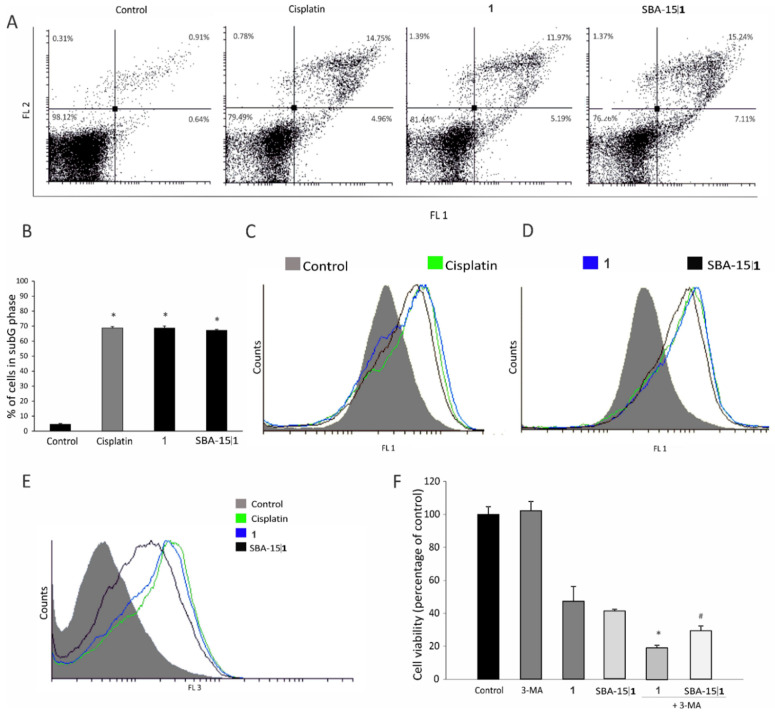
Effect of platinum(IV) conjugate **1** and corresponding MSNs SBA-15|**1** on 4T1 mouse breast cancer cells: (**A**) apoptosis (Annexin V-FITC/propidium iodide, (**B**) cell cycle—subG phase (* *p* < 0.05 refers to untreated cells), (**C**) caspase activation (Apostat), (**D**) cell proliferation potential (CFSE), (**E**) induction of autophagy (AO staining), and (**F**) effect of 3-MA addition on viability of 4T1 cells (MTT assay) (* *p* < 0.05 refers to culture treated with **1**, # *p* < 0.05 refers to culture treated with SBA-15|**1**).

Aiming to get an insight in the plausible mechanism of drug-induced cytotoxicity, flow cytometric evaluation of apoptotic cell death, cell cycle perturbation, rate of cell proliferation, caspase activation, and induction of autophagic response was employed upon treatment of the 4T1 cells with IC_50_ doses of **1** and cisplatin and an MC_50_ dose of SBA-15|**1**. The obtained results reveal that in all applied treatments, induction of massive apoptosis was in the background of diminished cell viability (Figure 5A). In concordance, significant accumulation of fragmented DNA in subG compartment of the cell cycle (Figure 5B), together with a strong caspase activation (Figure 5C) was found in cultures exposed to **1**, SBA-15|**1** and cisplatin. In parallel, surviving cell subpopulation lost the dividing potential (CFSE assay, Figure 5D). Since the process of autophagy is deeply involved in the cells’ response to toxic stimuli, varying from protective to destructive, it is very important to define the eventual presence of autophagosomes in cultures exposed to the experimental therapeutics [78,79].

An intensified autophagic process (AO assay, Figure 5E) in all treated cultures was noticed. However, additional decrease in cell viability was observed also upon neutralization of autophagy through concomitant exposure of the treated cells to the specific inhibitor 3-MA (Figure 5F). Thus, the obtained data suggest that autophagy has a protective role, defending the cell from destructive stimuli.

**Figure 6:**
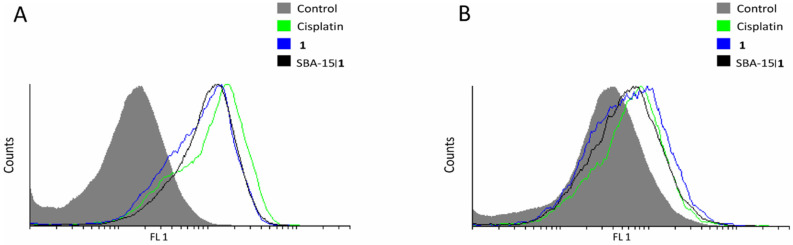
Effect of platinum(IV) conjugate **1** and corresponding MSNs SBA-15|**1** on 4T1 mouse breast cancer cells: (**A**) intracellular NO production (DAF-FM) and (**B**) ROS/RNS production (DHR).

In addition, treatment with conjugate **1** and SBA-15|**1** triggered increased production of nitric oxide (DAF-FM assay, Figure 6A), as well as ROS/RNS (DHR assay, Figure 6B).

Altogether, platinum(IV) conjugate **1**, free and loaded in SBA-15, clearly showed potential to downregulate cell growth through inhibition of proliferation and apoptotic cell induction which is even amplified with autophagy suppression. Behind the observed effect could be the enhanced production of reactive species which affect different cellular processes and subsequently the cell viability [80,81].

### 3.6. In Vivo Decrease of Breast Cancer Growth

**Figure 7:**
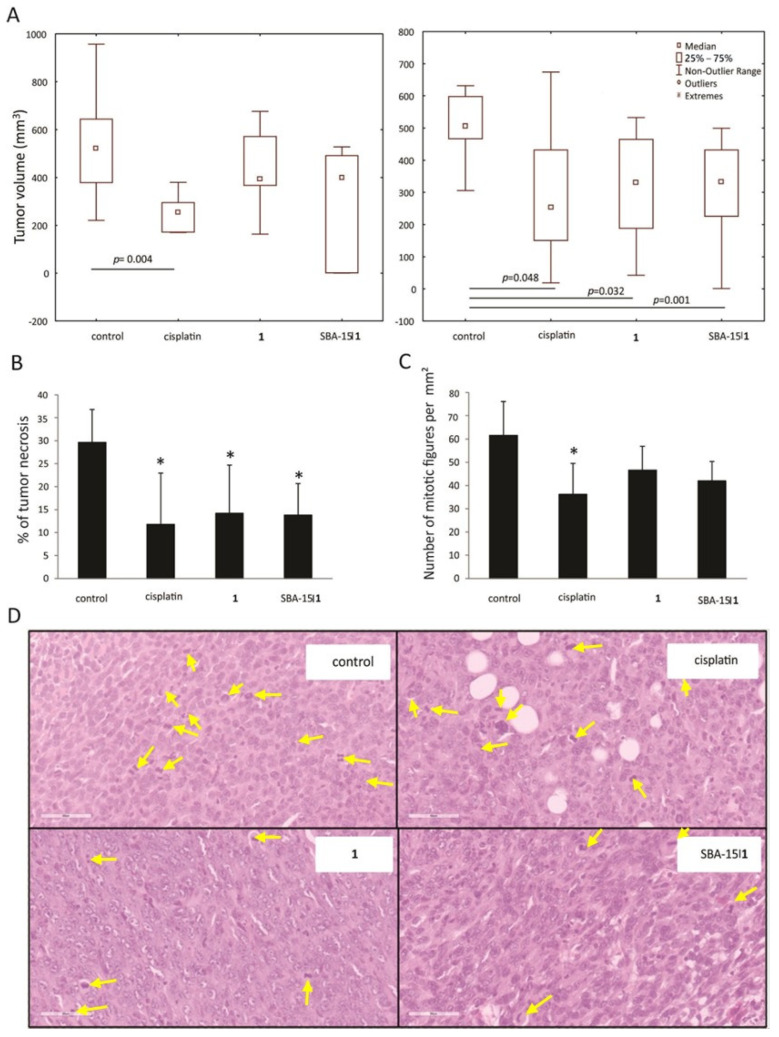
In vivo effect of platinum(IV) conjugate **1** and the corresponding MSNs SBA-15|**1** on 4T1-inoculated breast tumors: (**A**) tumors’ growth suppression, (**B**) tumor necrotic areas, (**C**) mitotic index, and (**D**) mitotic figures (marked with yellow arrows, H/E stain, 400× magnification).

With the aim to check the effectiveness of conjugate **1** and SBA-15|**1** in vivo, an orthotopic model of breast cancer was used. Tumors were induced by inoculation of 4T1 cells in fat pad of the fourth breast in syngeneic BALB/c mice, and treatment started when tumors became palpable. Cisplatin was applied in a therapeutic dose [82], while **1** or SBA-15|**1** were applied in an equimolar dose with regards to cisplatin. Results revealed approximately 50% tumor volume reduction upon treatment with cisplatin and SBA-15|**1**, while conjugate **1** alone decreased the tumor volume by 20% (Figure 7A). As statistical significance was not reached after the application of the mentioned doses, the experiment was repeated with a dose of 10 mg kg^−1^ for the free conjugate **1** and 35 mg kg^−1^ for SBA-15|**1**. The obtained results revealed remarkable inhibition of tumor growth in all three experimental groups (Figure 7B). The experiment was concluded with the sacrifice of the animals and histopathological examination of their tumors, liver and kidney.

Average percentage of tumor’s necrosis on tissue cross section was the largest in the control group (30%), while all treatments dramatically decreased necrotic area surface (conjugate **1** (14%), SBA-15|**1** (14%) and cisplatin (12%), Figure 7C). In addition, mitotic index, represented as mitotic figure count per one square millimeter, again was the highest in the control group, while rate of mitosis was diminished in tumor tissue sections of all treated groups (Figure 7B,D). Having in mind that tumors with large necrotic areas are recognized as highly aggressive and associated with poor prognosis [83], reduced necrotic zones together with lower mitotic rate upon the treatments indicates decreased neoplastic potential of the tumor tissue and underlines the advantage of the applied treatments.

**Figure 8:**
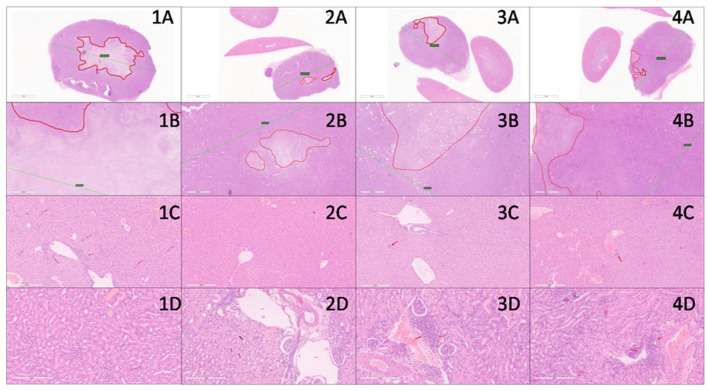
In vivo effect of platinum(IV) conjugate **1** and the corresponding MSNs SBA-15|**1** on 4T1-inoculated breast tumors—histopathological evaluation: tissues in columns 1, 2, 3, and 4 represent control, cisplatin, **1** and SBA-15|**1** groups, respectively. Tissues in rows **A** and **B** are tumor tissues (8× and 40× magnification, respectively) with red lines delineating necrotic areas and green lines denoting largest diameter of tumor. Row **C** is liver tissue with red arrows pointing to hematopoietic cells (40× magnification). Row **D** is kidney tissue (200× magnification). In picture **2D**, black arrows point to protein cast in kidney tubules and red arrows point to dilated blood vessels. Red arrows in **3D** and **4D** point to areas of inflammatory infiltrate.

In liver, findings characteristic for 4T1 metastatic breast cancer model with presence of extramedullary hematopoiesis, mainly represented by cells of myeloid lineage and in lesser amount by megakaryocytes, were observed [84,85]. Hematopoietic cells were present around and within the portal tracts, as well as in the sinusoid of the liver. The highest level of hematopoiesis was found in the control group, while upon treatment, in all treated groups it was almost absent (Figure 8). This fact led to the conclusion that all three treatments were effective in a way that inhibits hematopoiesis which was conditioned with the amount of tumor’s cell burden.

No relevant signs of hepatotoxicity were found in any of the groups. There was no inflammation nor necrosis in this experimental setting (Figure 8). Some degree of dilatation of small blood vessels (mainly venules) mostly in the SBA-15|**1** group, without knowledge of functional derangement, was noted.

In kidney tissue, there were no significant signs of nephrotoxicity in all groups. Normal histomorphology was dominantly registered (Figure 8). Concordantly, all parameters (blood, bilirubin, pH, ketone, protein, specific gravity, leukocyte, urobilinogen and nitrite) tested in urine of animals which were exposed to treatment, showed no difference to the control group of untreated animals (ESI, Table S4). In the group treated with cisplatin, however, protein casts were found in the tubules, and dilatation of small periglomerular blood vessels was observed. This indicates that despite the fact that previous findings suggested the absence of toxicity, cisplatin has a nephrotoxic input [86] which was not observed for **1** and SBA-15|**1**.

## 4. Conclusions

In summary, we report on three cisplatin derivatives bearing acetyl-protected caffeate (**1**), acetyl-protected ferulate (**2**) or ferulate (**3**) as axial ligands. Upon loading of these platinum(IV) complexes into mesoporous silica SBA-15 material, the MSNs SBA-15|**1**, SBA-15|**2** and SBA-15|**3** were prepared. Under simulated physiological settings, the MSNs maintained their structural integrity, enabling a moderate (for SBA-15|**3**) to extremely slow release (for SBA-15|**1** and SBA-15|**2**) of the appropriate platinum(IV) complex. This delayed release is particularly advantageous in vivo because it results in low drug release during circulation and longer drug exposure upon accumulation in the tumor.

The free platinum(IV) conjugates **1**–**3** demonstrated higher or similar antiproliferative activity with respect to cisplatin in four human breast cancer cell lines (BT-474, MCF-7, MDA-MB-468 and HCC1937). Great anticancer potential was, however, observed for all three complexes upon their loading into the nanostructured SBA-15. Thus, SBA-15|**1**, SBA-15|**2** and SBA-15|**3** demonstrated significantly higher cytotoxicity as compared to cisplatin with nanomolar IC_50_ values in all four cell lines.

For the most potent complex **1** and the corresponding SBA-15|**1**, mechanistic investigations made in the mouse breast cancer cell line 4T1 indicated the potential of drug and nanomaterial to downregulate cell growth through inhibition of proliferation and apoptotic cell induction. Drug toxicity can be further amplified via autophagy suppression. All observed effects could be due to the enhanced production of NO and ROS/RNS, which affect different cellular processes and, consequently, the cell viability. Furthermore, **1** and SBA-15|**1**, caused in vivo inhibition of tumor growth in BALB/c mice, decreasing the necrotic area and lowering the mitotic rate. Importantly, nephrotoxicity present in mice treated with cisplatin, was not observed when **1** or SBA-15|**1** were used.

## Figures and Tables

**Table 1:** Selected physical parameters of the MSNs.

MSNs	*S*BET	Wall Thickness	Pore Diameter	Pore Volume	Lattice Parameter	XRD	
	[m^2^ g^−1^]	[nm]	[nm]	[cm^3^ g^−1^]	*a* [nm]	2*θ* [°]	*hkl*
SBA-15 [48]	517	3.66	4.74	0.72	8.4	1.0802	100
						1.8032	111
						2.0645	200
SBA-15|1	245	3.34	4.75	0.34	8.1	1.1404	100
						1.8543	111
						2.1378	200
SBA-15|2	260	4.26	4.46	0.37	8.7	1.0311	100
						1.7450	111
						2.0075	200
SBA-15|3	292	5.03	3.68	0.37	8.7	1.0149	100
						1.7603	111
						2.0228	200

**Table 2:** IC_50_ [μM] and MC_50_ [μg mL^−1^] values of cisplatin, **1**−**3**, free ligands, SBA-15, and SBA-15|**1**−SBA-15|**3** determined with CV and MTT assays (72 h).

Compound/Material	CV					MTT				
	MDA-MB-468	HCC1937	MCF-7	BT-474	4T1	MDA-MB-468	HCC1937	MCF-7	BT-474	4T1
	IC_50_ [µM]					IC_50_ [µM]				
Acetyl-protected caffeic acid	>100					>100				
Acetyl-protected ferulic acid	>100					>100				
Ferulic acid	>100					>100				
Cisplatin	3.3 ± 0.2	7.6 ± 0.9	33.6 ± 4.8	54.9 ± 6.0	3.2 ± 0.3	0.6 ± 0.1	4.3 ± 0.7	32.0 ± 4.3	70.3 ± 8.5	1.3 ± 0.1
Cisplatin-acetyl protected caffeate conjugate (**1**)	2.6 ± 0.7	8.9 ± 0.3	16.0 ± 2.3	5.4 ± 0.3	4.4 ± 0.7	2.7 ± 0.8	9.9 ± 0.5	> 50	4.5 ± 0.4	3.3 ± 0.4
Cisplatin-acetyl protected ferulate conjugate (**2**)	3.5 ± 0.5	10.4 ± 1.1	8.4 ± 2.2	5.9 ± 0.4		3.8 ± 0.8	5.5 ± 4.3	27.6 ± 2.6	9.7 ± 1.2	
Cisplatin-ferulate conjugate (**3**)	7.1 ± 0.3	11.1 ± 0.8	19.9 ± 1.3	>50		8.3 ± 0.7	15.1 ± 2.1	>50	>50	
SBA-15|**1** ^(^^a)^	0.3 ± 0.03	1.7 ± 0.1	1.6 ± 0.1	0.0001 ± 0.00001	1.0 ± 0.2	0.1 ± 0.001	0.3 ± 0.02	0.3 ± 0.1	0.1 ± 0.001	0.8 ± 0.04
SBA-15|**2** ^(a)^	0.1 ± 0.01	1.0 ± 0.1	1.0 ± 0.1	0.0017 ± 0.0001		0.02 ± 0.002	0.2 ± 0.02	/	0.1 ± 0.02	
SBA-15|**3** ^(a)^	1.6 ± 0.1	2.6 ± 0.5	4.4 ± 0.5	0.7 ± 0.1		0.4 ± 0.01	1.0 ± 0.2	3.7 ± 0.2	1.3 ± 0.2	
	**MC_50_ [µg mL^−1^]**					**MC_50_ [µg mL^−1^]**				
SBA-15	>100					>100				
SBA-15|**1**	3.0 ± 0.5	19.9 ± 1.0	18.5 ± 1.0	0.0017 ± 0.0002	11.9 ± 1.9	0.6 ± 0.02	3.3 ± 0.3	7.3 ± 0.8	0.7 ± 0.02	9.7 ± 0.5
SBA-15|**2**	3.6 ± 0.4	32.9 ± 2.1	35.0 ± 3.0	0.6 ± 0.006		0.6 ± 0.1	6.5 ± 0.7	>50	4.3 ± 0.7	
SBA-15|**3**	3.9 ± 0.2	6.4 ± 1.2	10.7 ± 1.3	1.7 ± 0.3		1.1 ± 0.03	2.3 ± 0.4	5.0 ± 0.5	3.3 ± 0.6	

^(a)^ recalculated based on platinum content (EDX) and drug release (ICP-OES).

## Data Availability

Not applicable.

## Supplementary Materials

The following supporting information can be downloaded at https://www.mdpi.com/article/10.3390/nano12213767/s1. Figure S1. ^1^H NMR spectrum of **1** in DMSO-d_6_; Figure S2. ^13^C{^1^H} NMR spectrum of **1** in DMSO-*d*_6_; Figure S3. ^195^Pt{^1^H} NMR spectrum of **1** in DMSO-*d*_6_; Figure S4. HR-ESI-MS (positive mode, CH_3_OH) of **1**, *m*/*z* [2M+H]^+^; Figure S5. HR-ESI-MS (positive mode, CH_3_OH) of **1**, *m*/*z* [M+H]^+^; Figure S6. ^1^H NMR spectrum of **2** in DMSO-*d*_6_; Figure S7. ^13^C{^1^H} NMR spectrum of **2** in DMSO-*d*_6_; Figure S8. ^195^Pt{^1^H} NMR spectrum of **2** in DMSO-*d*_6_; Figure S9. HR-ESI-MS (positive mode, CH_3_OH) of **2**, *m*/*z* [2M+H]^+^; Figure S10. HR-ESI-MS (positive mode, CH_3_OH) of **2**, *m*/*z* [M+H]^+^; Figure S11. HR-ESI-MS (positive mode, CH_3_OH) of **2**, *m*/*z* [M+Na]^+^; Figure S12. ^1^H NMR spectrum of **3** in DMSO-*d*_6_; Figure S13. ^13^C{^1^H} NMR spectrum of **3** in DMSO-*d*_6_; Figure S14. ^195^Pt{^1^H} NMR spectrum of **3** in DMSO-*d*_6_; Figure S15. HR-ESI-MS (positive mode, CH_3_OH) of **3**, *m*/*z* [M+H]^+^; Figure S16. HR-ESI-MS (positive mode, CH_3_OH) of **3**, m/z [2M+H]^+^; Figure S17. HR-ESI-MS (positive mode, CH_3_OH) of **3**, *m*/*z* [2M+Na]^+^; Figure S18. HR-ESI-MS (positive mode, CH_3_OH) of **3**, *m*/*z* [2M+K]^+^; Figure S19. Attempted *O*-deacetylation of **1** with enzyme Amano lipase A from *Aspergillus niger*; time-resolved ^1^H NMR spectra in DMSO-*d*_6_, aromatic region section; Figure S20. Stability of **1** in DMSO-*d*_6_ over 72 h; time-resolved ^1^H NMR spectra; Figure S21. Stability of **2** in DMSO-*d*_6_ over 72 h; time-resolved ^1^H NMR spectra; Figure S22 Stability of **3** in DMSO-*d*_6_ over 72 h; time-resolved ^1^H NMR spectra; Figure S23. Spatial distribution of silicon and platinum in SBA 15|**1**, SBA 15|**2** and SBA 15|**3** determined through EDX mapping; Figure S24. Zero order, first order, Higuchi and Korsmeyer-Peppas kinetic release of **1** from SBA-15|**1**; Figure S25. Zero order, first order, Higuchi and Korsmeyer-Peppas kinetic release of **2** from SBA-15|**2**; Figure S26. Zero order, first order, Higuchi and Korsmeyer-Peppas kinetic release of **3** from SBA-15|**3**; Figure S27. Cell viability of cisplatin, **1**, **2** and **3** determined by CV and MTT assays in MDA-MB-468 and HCC1937 human breast cancer cell lines; Figure S28. Cell viability of cisplatin, **1**, **2** and **3** determined by CV and MTT assays in MCF-7 and BT-474 human breast cancer cell lines; Figure S29. Cell viability of SBA-15|**1**, SBA-15|**2** and SBA-15|**3** determined by CV and MTT assays in MDA-MB-468 and HCC1937 human breast cancer cell lines; Figure S30. Cell viability of SBA-15|**1**, SBA-15|**2** and SBA-15|**3** determined by CV and MTT assays in MCF-7 and BT-474 human breast cancer cell lines; Figure S31. Cell viability of cisplatin, **1** and SBA-15|**1** determined by CV and MTT assays in mouse-derived 4T1 breast cancer cell line; Table S1. Relative weight % of silicon and platinum at six points in SBA-15|**1**, SBA-15|**2** and SBA-15|**3** determined by EDX analysis; Table S2. Platinum load (wt%) and encapsulation efficiency (%) in SBA-15|**1**, SBA-15|**2** and SBA-15|**3**; Table S3. Constants and coefficient of determinations (R^2^) for each model; Table S4. Urine parameters of untreated animals (control) and animals exposed to treatment with cisplatin, **1** and SBA-15|**1**.
