# Supplementary material for: Mesoporous Silica Nanoparticles Enhance the Anticancer Efficacy of Platinum(IV)-Phenolate Conjugates in Breast Cancer Cell Lines
Source: Nanomaterials (Basel). 2022 Oct 26;12(21):3767. doi: 10.3390/nano12213767 (PMC9659259; doi:10.3390/nano12213767)
Supplement: Supplementary file 1 [file nanomaterials-12-03767-s001.zip › nanomaterials-1960585-supplementary.pdf]

Mesoporous Silica Nanoparticles Enhance the Anticancer Efficacy of Platinum(IV)-Phenolate Conjugates in Breast Cancer Cell Lines

Ivana Predarska [1,2], Mohamad Saoud [3], Dijana Drača [4], Ibrahim Morgan [3], Teodora Komazec [4], Thomas Eichhorn [2], Ekatarina Mihajlović [4], Duško Dunđerović [5], Sanja Mijatović [4], Danijela Maksimović-Ivanić [4], Evamarie Hey-Hawkins [1,*] and Goran N. Kaluđerović [2,3,*]

[1] Faculty of Chemistry and Mineralogy, Institute of Inorganic Chemistry, Universität Leipzig, Johannisallee 29, 04103 Leipzig, Germany
[2] Department of Engineering and Natural Sciences, University of Applied Sciences Merseburg, Eberhard-Leibnitz-Str. 2, 06217 Merseburg, Germany
[3] Department of Bioorganic Chemistry, Leibniz Institute of Plant Biochemistry, Weinberg 3, 06120 Halle (Saale), Germany
[4] Institute for Biological Research "Siniša Stanković", National Institute of Republic of Serbia, University of Belgrade; Bulevar despota Stefana 142, 11060 Belgrade, Serbia
[5] Institute of Pathology, School of Medicine, University of Belgrade, dr Subotića 1, 11000 Belgrade, Serbia
* Correspondence: hey@uni-leipzig.de (E.H.-H.); goran.kaluderovic@hs-merseburg.de (G.N.K.); Tel.: +49-341-97-36151 (E.H.-H.); +49-3461-46-2012 (G.N.K.).

Electronic Supplementary Information

Table of Contents

Characterization of platinum(IV) conjugates 1–3 3

NMR spectra of conjugate **1** 3

Mass spectra of conjugate **1** 5

NMR spectra of conjugate **2** 6

Mass spectra of conjugate **2** 8

NMR spectra of conjugate **3** 10

Mass spectra of conjugate 3........ 11

Attempted *O*-deacetylation of 1 with enzyme Amano lipase A 13

Stability of conjugates 1–3 in DMSO 14

Encapsulation efficiency calculation 16

Drug release kinetics 18

Cell viability 21

Urine parameters upon *in vivo* treatment 25

Characterization of platinum(IV) conjugates 1–3

NMR spectra of conjugate **1**

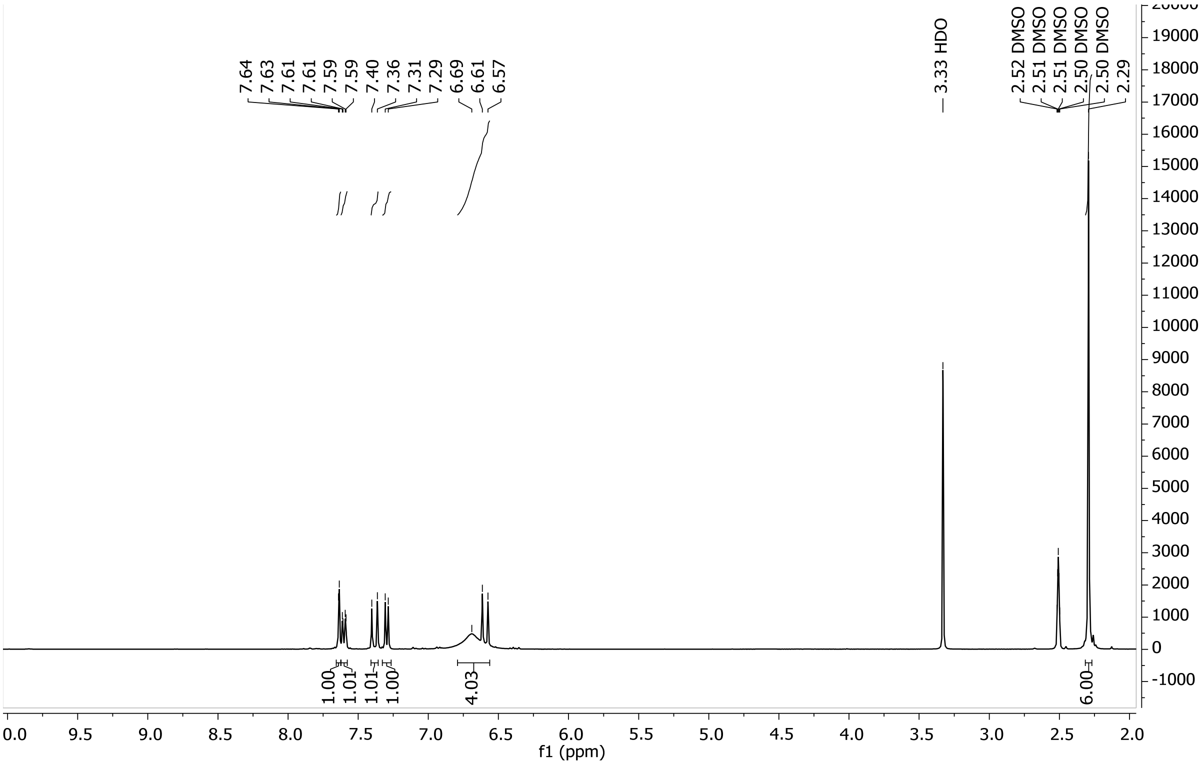

Figure S1. ^{1}H NMR spectrum of **1** in DMSO-d_6.

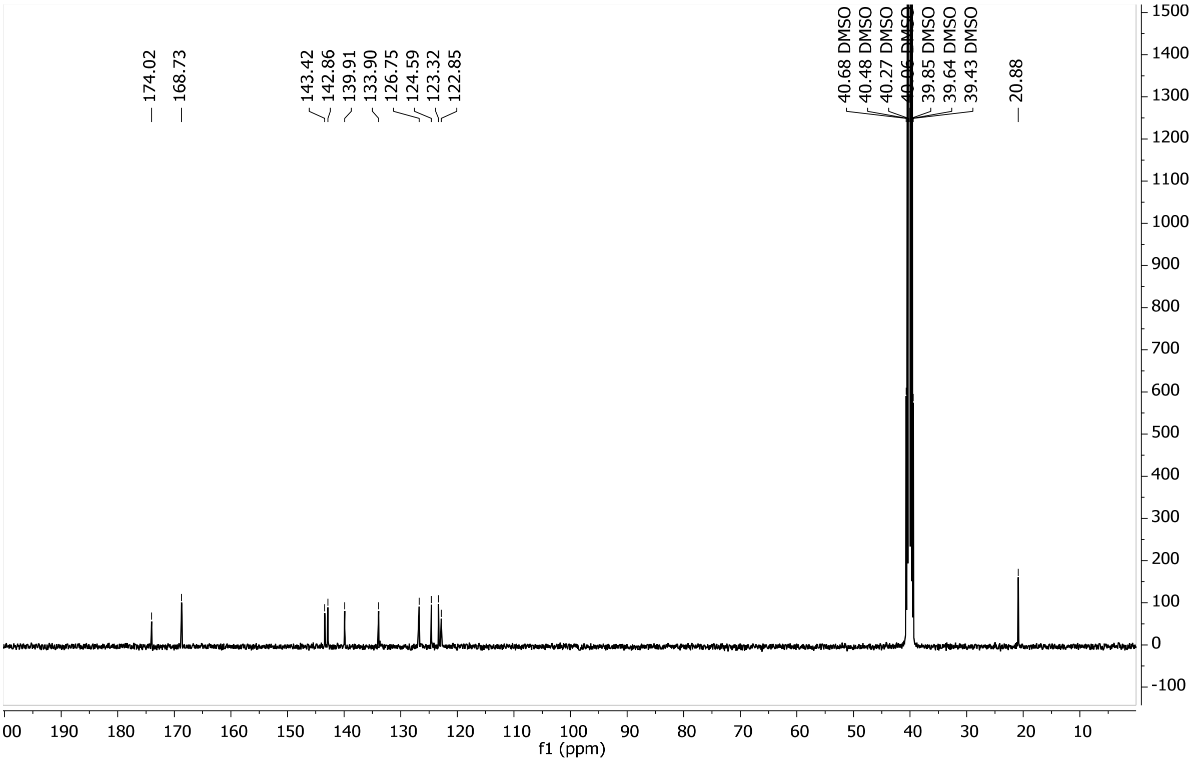

Figure S2. $^{13}C\{^{1}H\}$ NMR spectrum of **1** in DMSO-d_6.

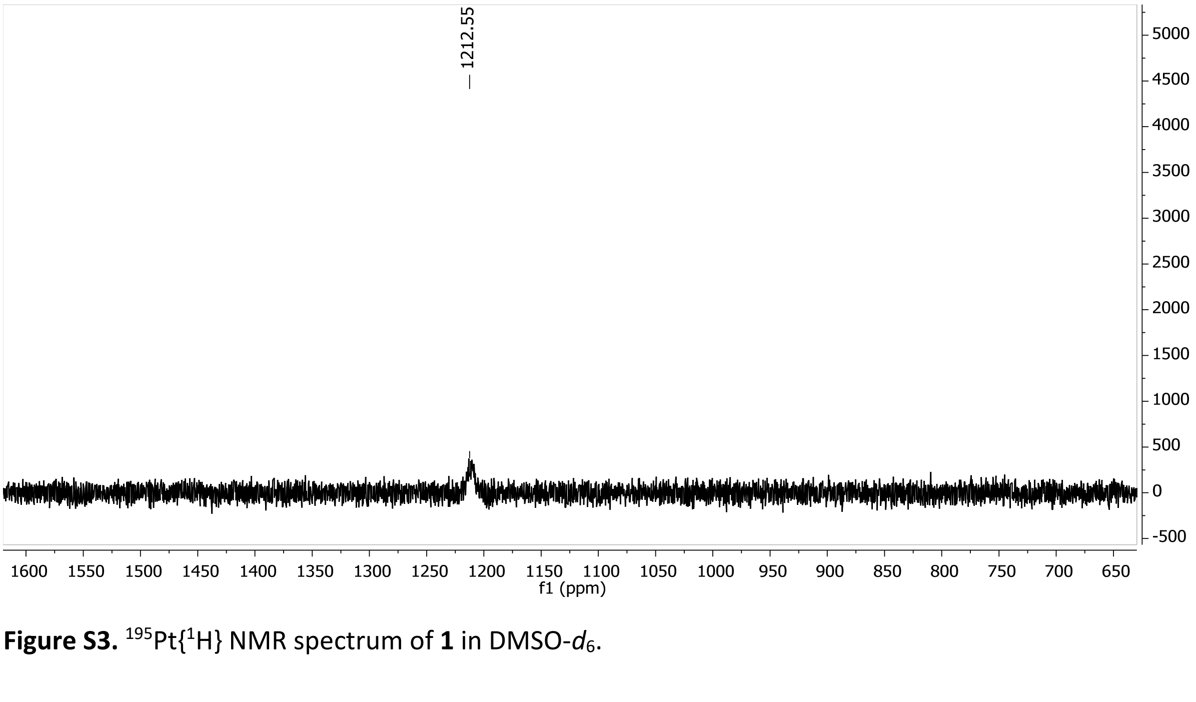

Figure S3. $^{195}Pt\{^{1}H\}$ NMR spectrum of **1** in DMSO-d_6.

Mass spectra of conjugate 1

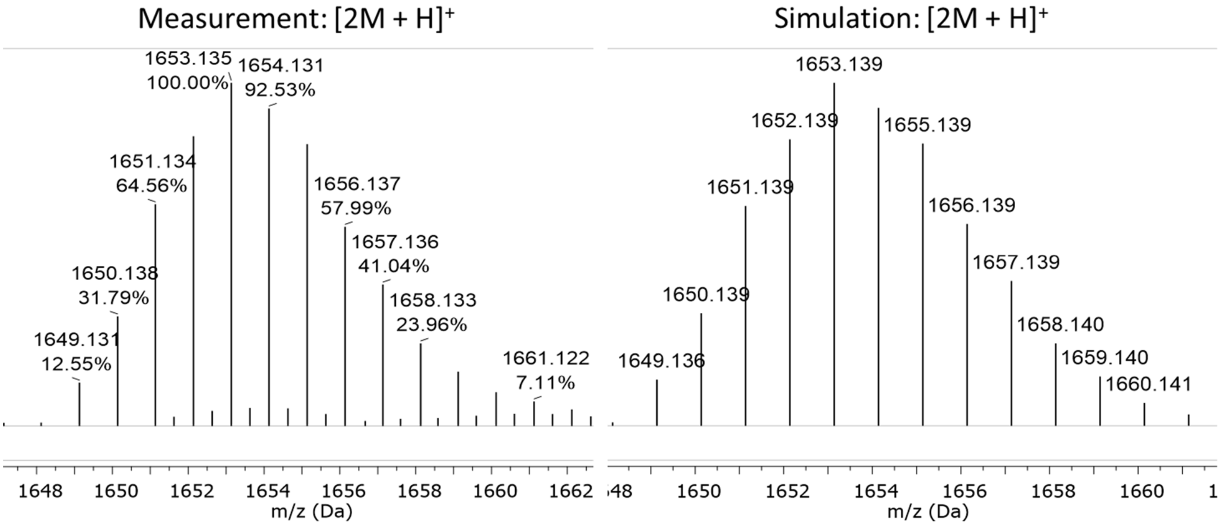

Figure S4. HR-ESI-MS (positive mode, CH_3OH) of **1**, *m/z* $[2M+H]^+$.

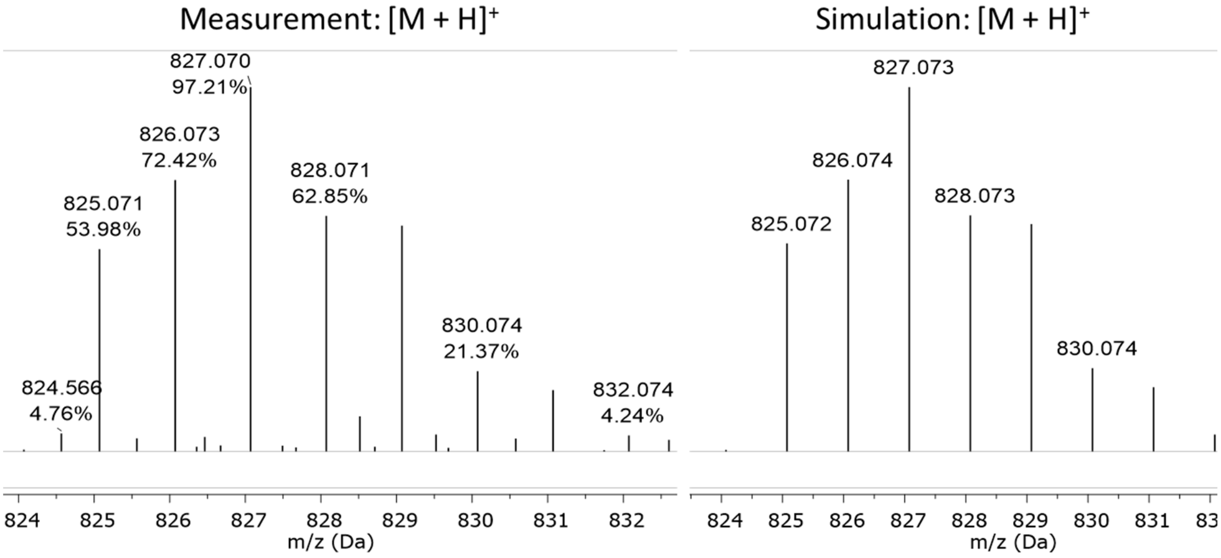

Figure S5. HR-ESI-MS (positive mode, CH_3OH) of **1**, *m/z* $[M+H]^+$.

NMR spectra of conjugate 2

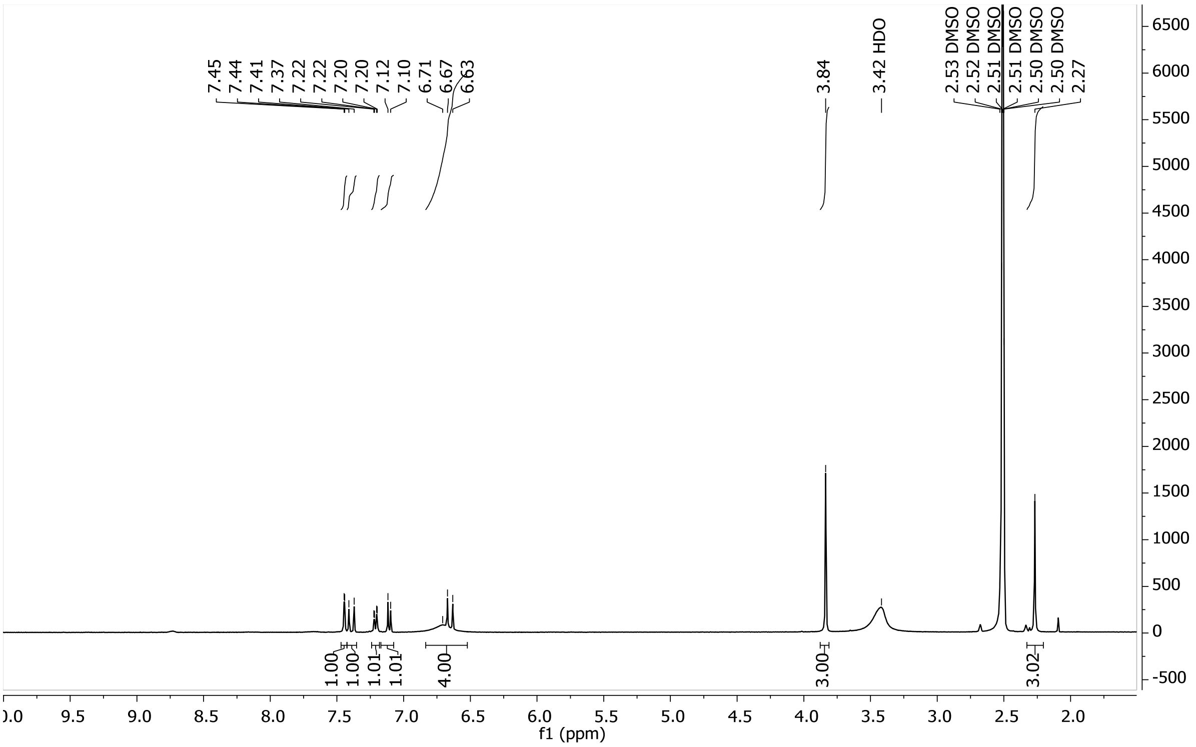

Figure S6. ^{1}H NMR spectrum of **2** in DMSO-d_6.

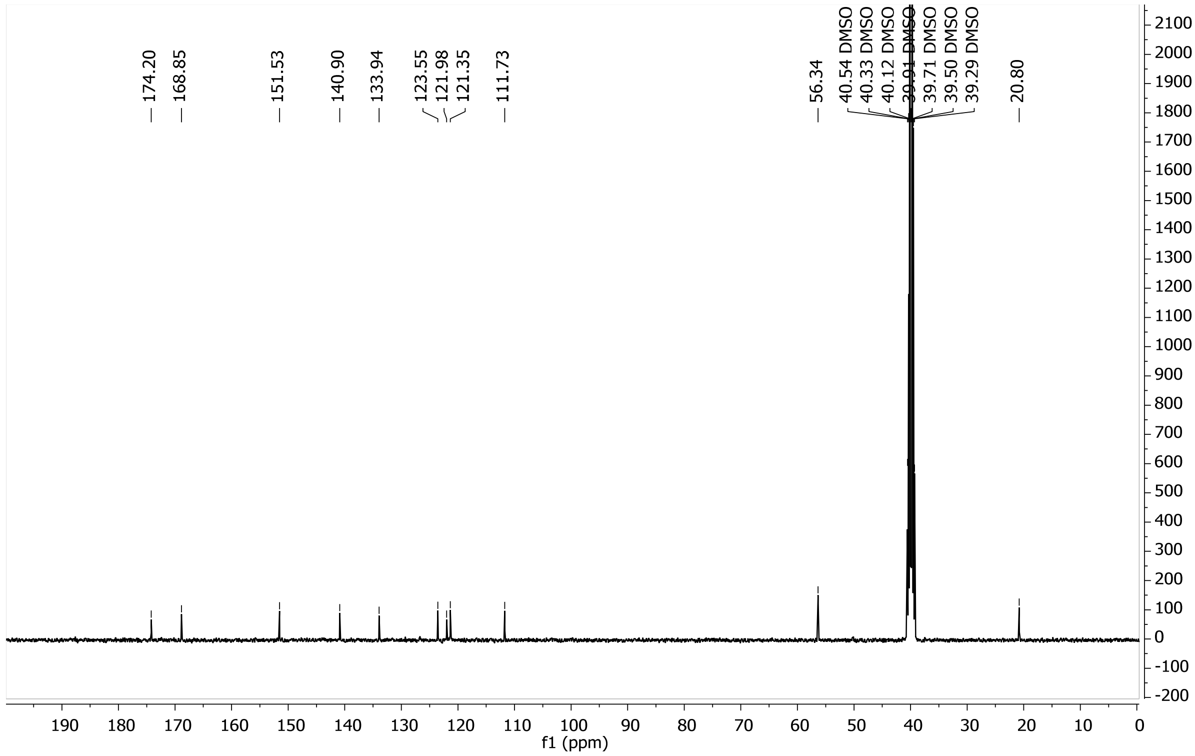

Figure S7. $^{13}C\{^{1}H\}$ NMR spectrum of **2** in DMSO-d_6.

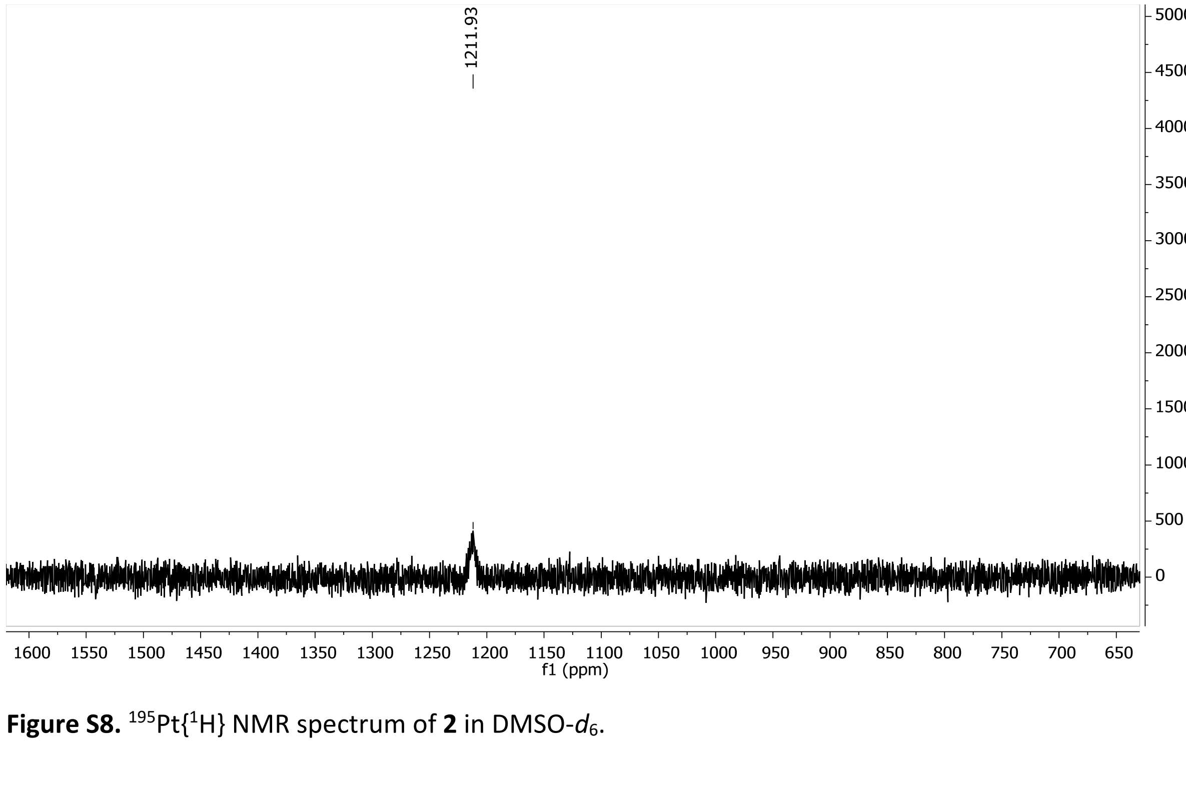

Figure S8. $^{195}Pt\{^{1}H\}$ NMR spectrum of **2** in DMSO-d_6.

Mass spectra of conjugate 2

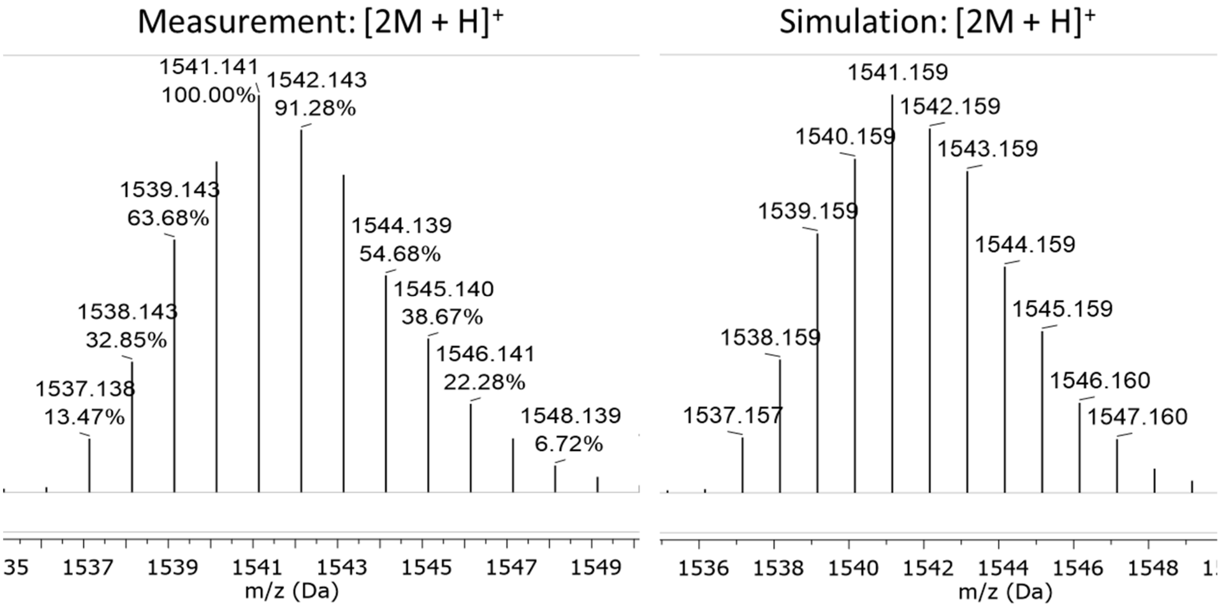

Figure S9. HR-ESI-MS (positive mode, CH_3OH) of **2**, *m/z* $[2M+H]^+$.

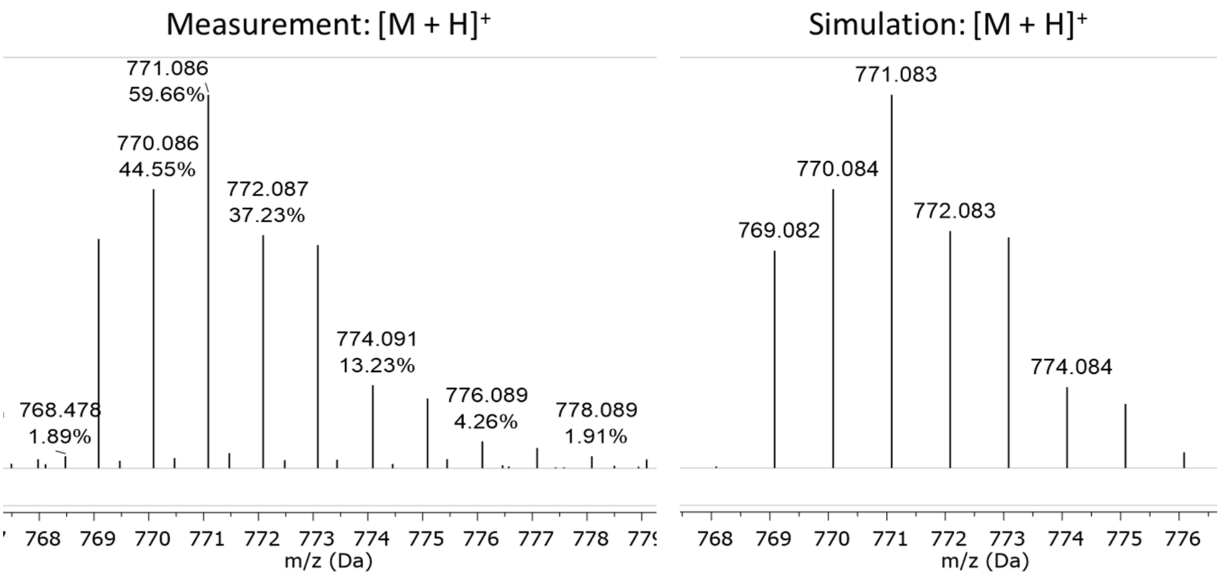

Figure S10. HR-ESI-MS (positive mode, CH_3OH) of **2**, *m/z* $[M+H]^+$.

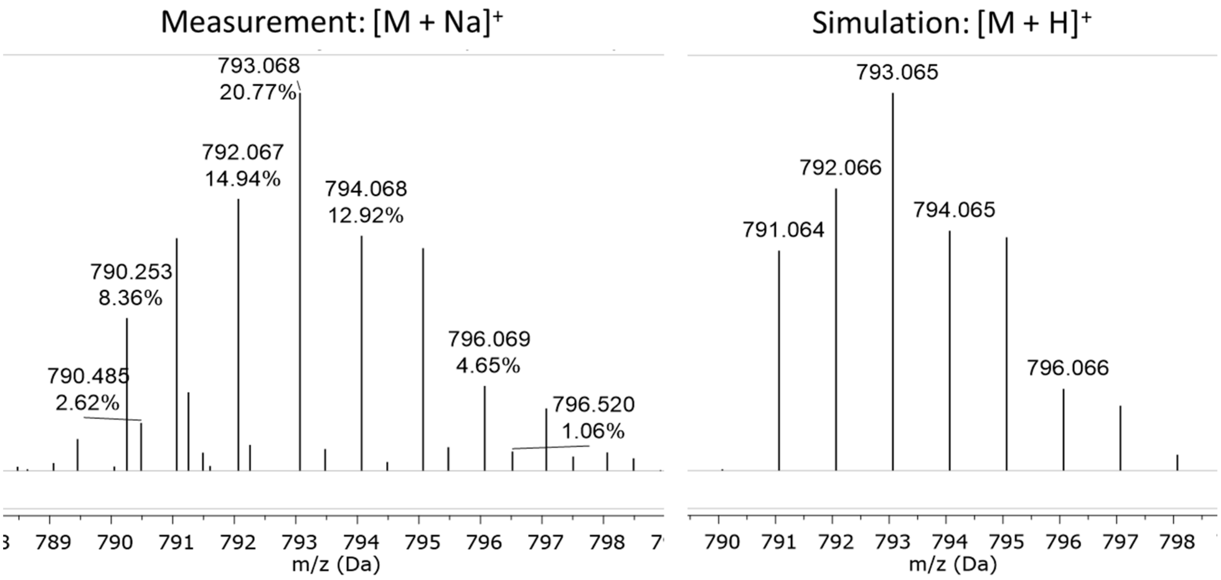

Figure S11. HR-ESI-MS (positive mode, CH_3OH) of **2**, *m/z* $[M+Na]^+$.

NMR spectra of conjugate **3**

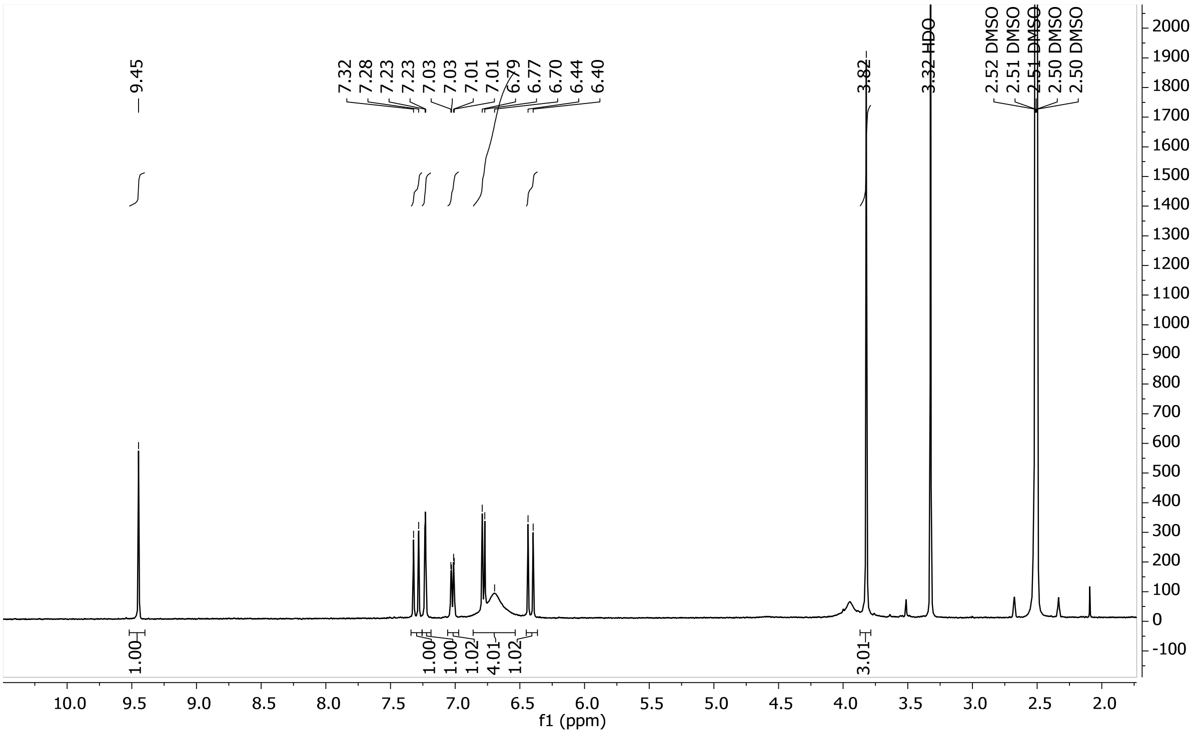

Figure S12. ^{1}H NMR spectrum of **3** in DMSO-d_6.

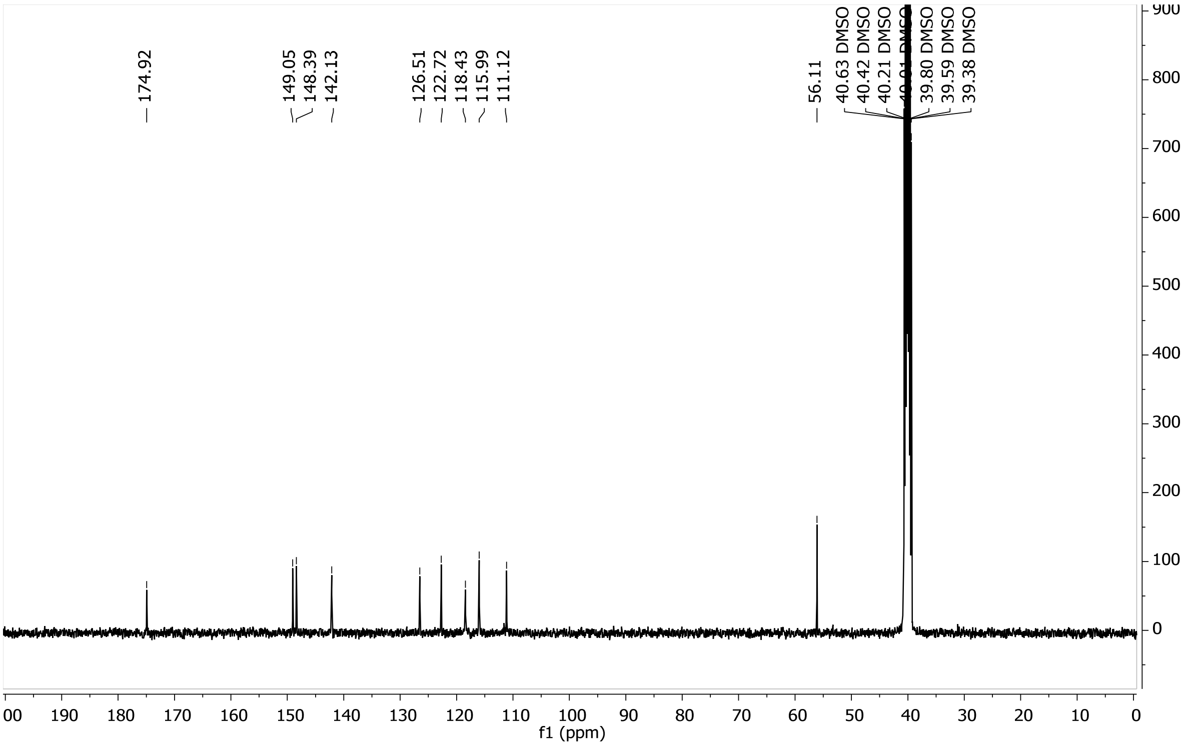

Figure S13. $^{13}C\{^{1}H\}$ NMR spectrum of **3** in DMSO-d_6.

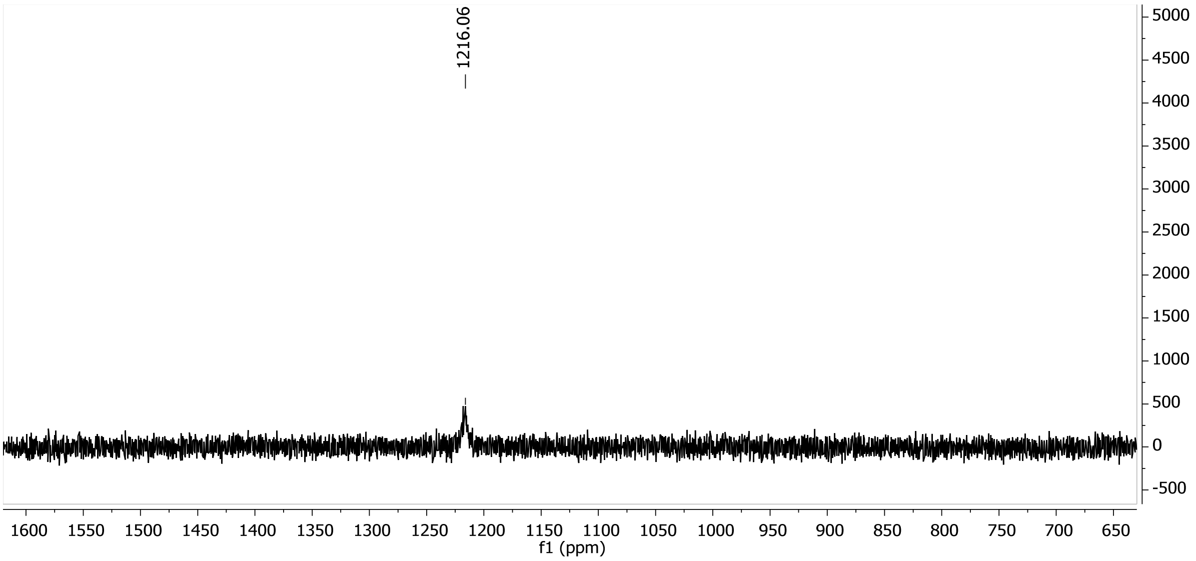

Figure S14. $^{195}Pt\{^{1}H\}$ NMR spectrum of **3** in DMSO-d_6.

Mass spectra of conjugate 3

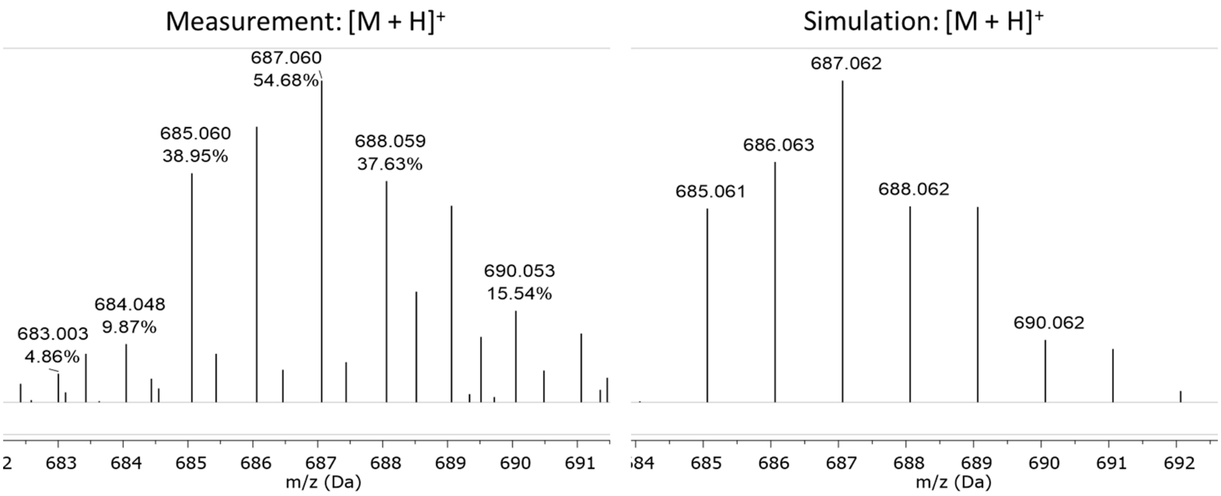

Figure S15. HR-ESI-MS (positive mode, CH_3OH) of **3**, *m/z* $[M+H]^+$.

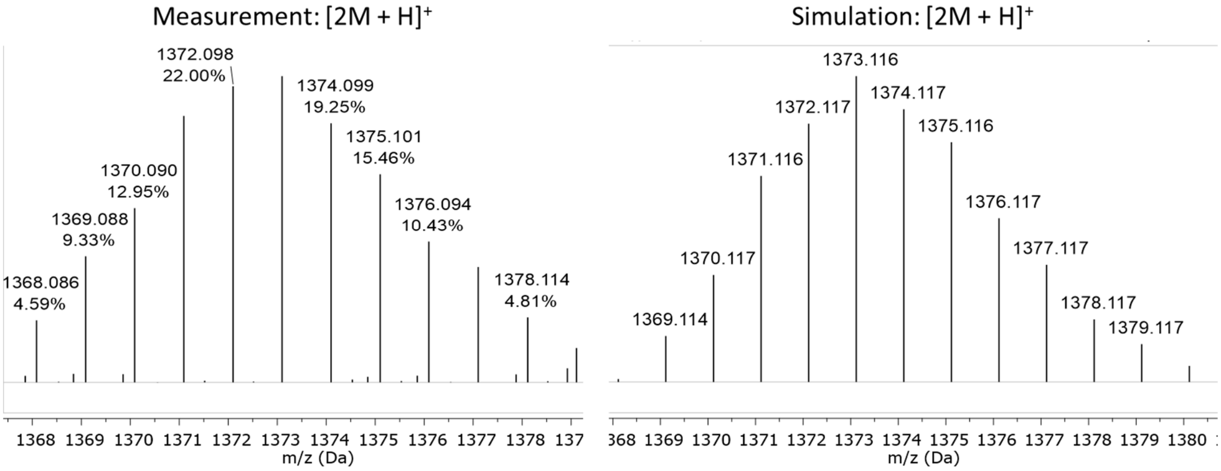

Figure S16. HR-ESI-MS (positive mode, CH_3OH) of **3**, *m/z* $[2M+H]^+$.

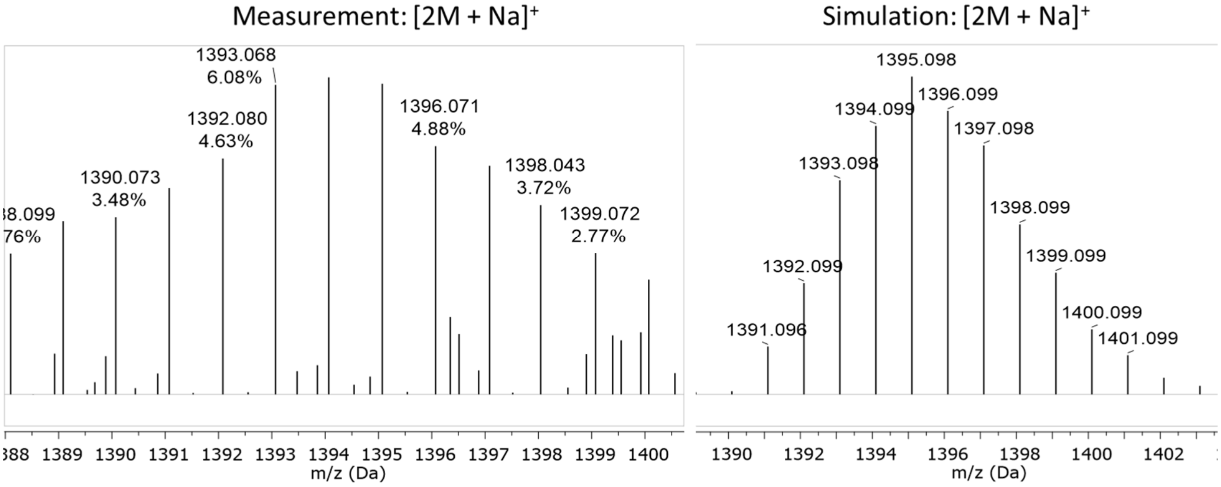

Figure S17. HR-ESI-MS (positive mode, CH_3OH) of **3**, *m/z* $[2M+Na]^+$.

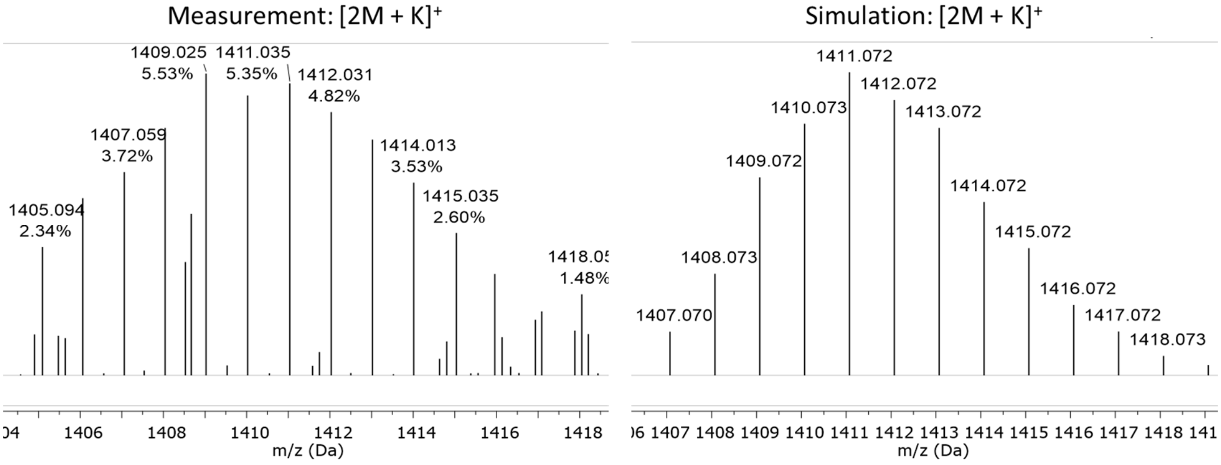

Figure S18. HR-ESI-MS (positive mode, CH_3OH) of **3**, *m/z* $[2M+K]^+$.

Attempted *O*-deacetylation of 1 with enzyme Amano lipase A

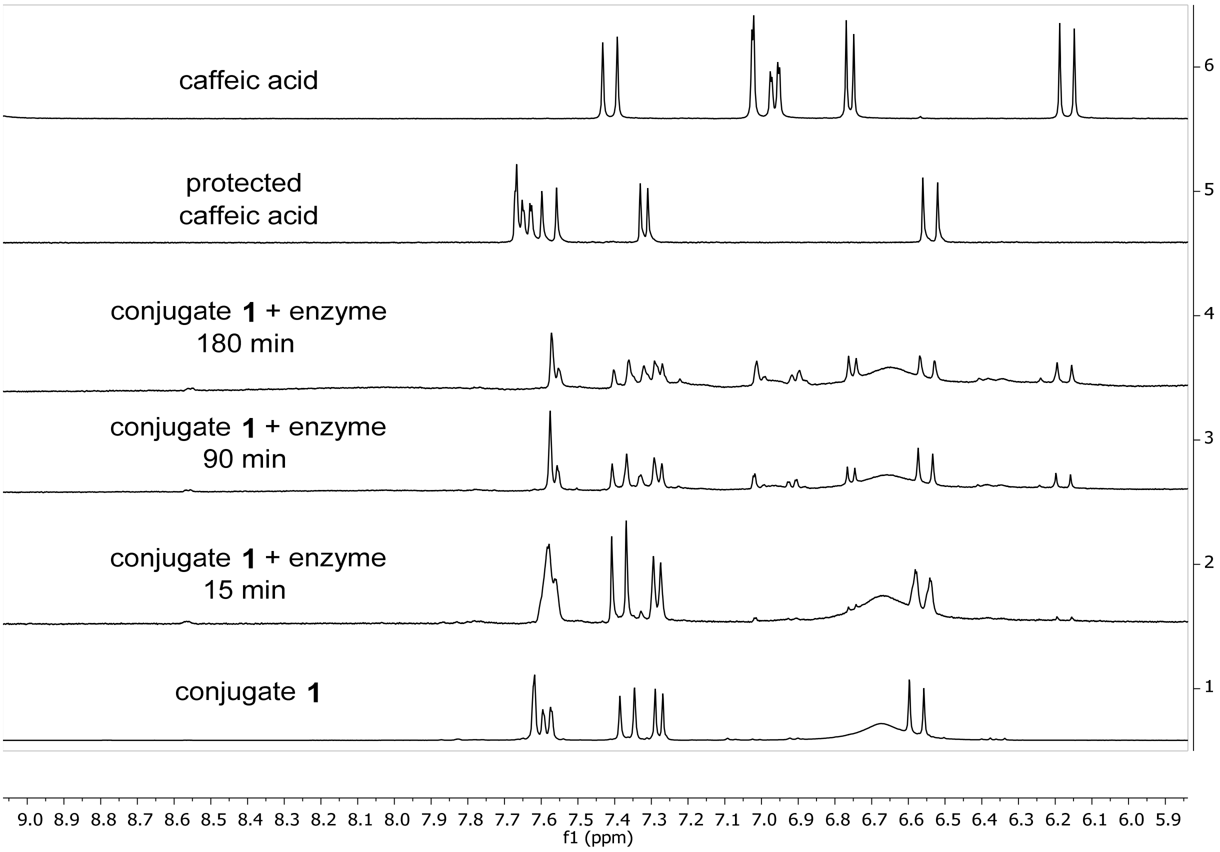

Figure S19. Attempted *O*-deacetylation of **1** with enzyme Amano lipase A from *Aspergillus niger*; time-resolved ^1H NMR spectra in DMSO-d_6, aromatic region section.

Stability of conjugates 1–3 in DMSO

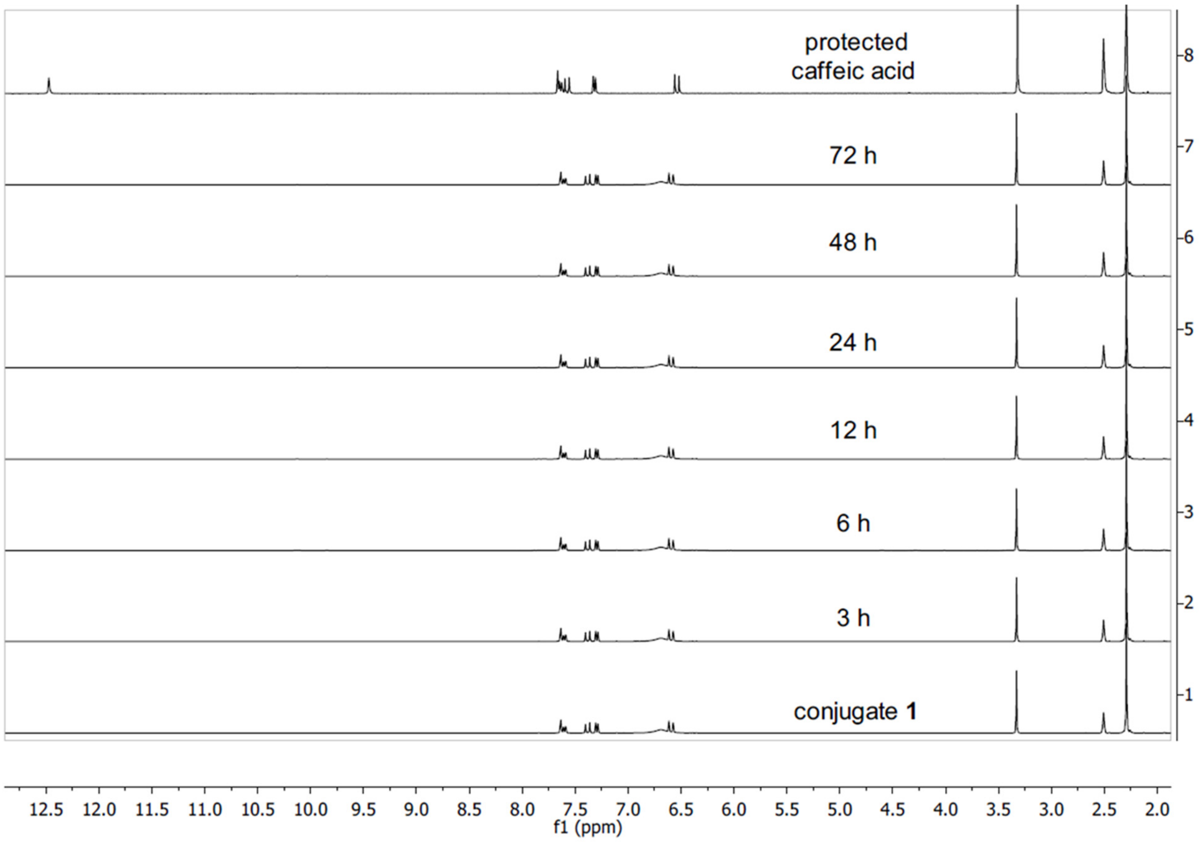

Figure S20. Stability of **1** in DMSO-d_6 over 72 h; time-resolved ^{1}H NMR spectra.

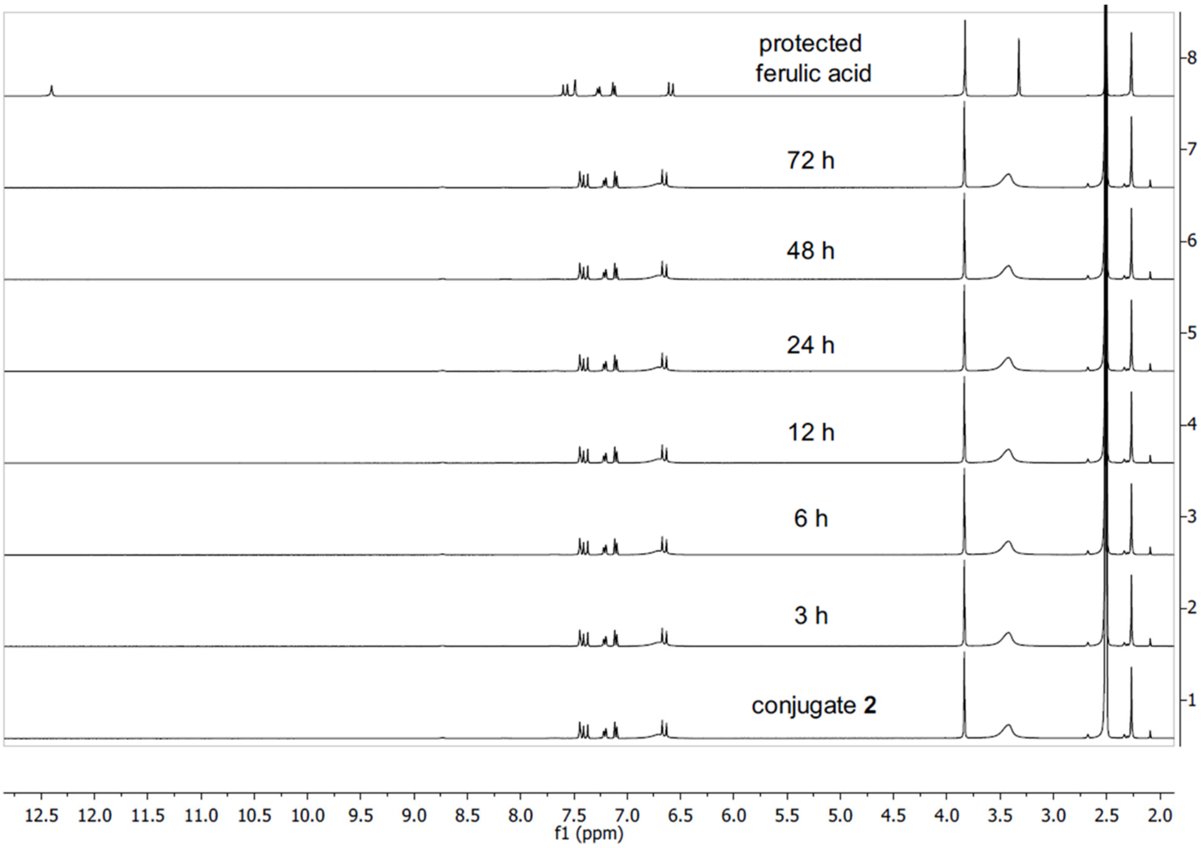

Figure S21. Stability of **2** in DMSO-d_6 over 72 h; time-resolved ^{1}H NMR spectra.

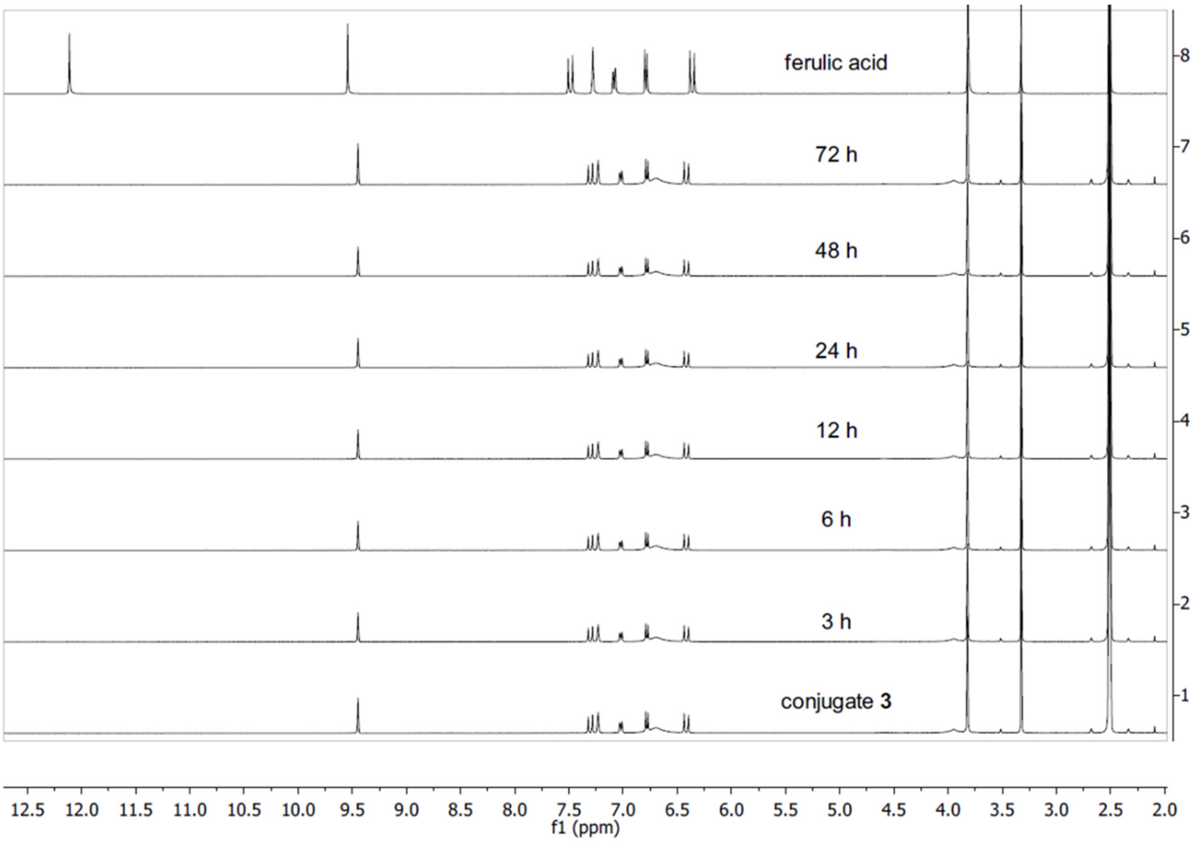

Figure S22. Stability of **3** in DMSO-d_6 over 72 h; time-resolved ^{1}H NMR spectra.

Encapsulation efficiency calculation

The encapsulation efficiency of the platinum(IV) conjugates **1**–**3** in the corresponding mesoporous silica nanoparticles (MSNs), SBA-15|**1**, SBA-15|**2** and SBA-15|**3**, was calculated based on the amount of platinum in the drug-loaded MSNs determined by energy dispersive X-ray (EDX) analysis. First, homogeneous distribution of the drugs (**1**, **2** and **3**) in the corresponding materials was established by determination of the spatial distribution of silicon and platinum accomplished through EDX mapping (Figure S23). Second, the relative weight % of silicon and platinum was measured at six randomly chosen points (Table S1). Based on these results, Pt/Si ratios were calculated for all materials (0.29, 0.35 and 0.37 for SBA-15|**1**, SBA-15|**2** and SBA-15|**3**, respectively, Table S2) and compared to the theoretical ones to afford the load content of platinum (7.51 wt%, 9.23 wt% and 10.33 wt% for SBA-15|**1**, SBA-15|**2** and SBA-15|**3**, respectively, Table S2) and the corresponding encapsulation efficiency (70.36 %, 83.83 % and 89.24 % for SBA-15|**1**, SBA-15|**2** and SBA-15|**3**, respectively, Table S2).

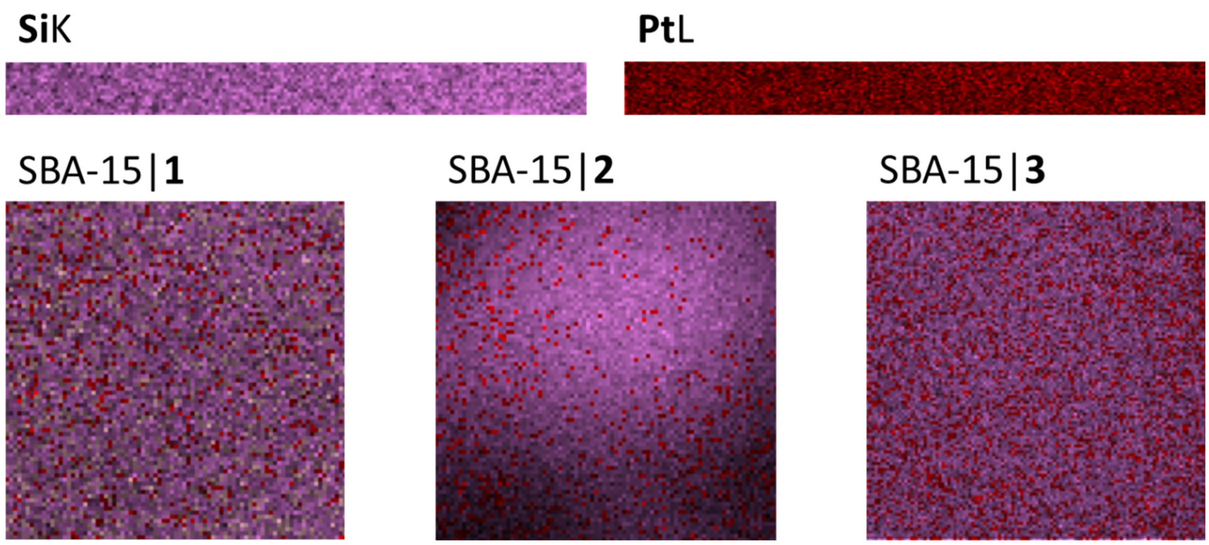

Figure S23. Spatial distribution of silicon and platinum in SBA 15|**1**, SBA 15|**2** and SBA 15|**3** determined through EDX mapping.

Table S1. Relative weight % of silicon and platinum at six points in SBA-15|**1**, SBA-15|**2** and SBA-15|**3** determined by EDX analysis.

	SBA-15|**1**		SBA-15|**2**		SBA-15|**3**	
	**Si**	**Pt**	**Si**	**Pt**	**Si**	**Pt**
	wt%					
	78.2	21.8	70.4	29.6	73.5	26.5
	76.3	23.7	73.2	26.8	74.1	25.9
	76.4	23.6	76.8	23.2	72.2	27.8
	77.6	22.4	72.1	27.9	72.4	27.6
	77.9	22.1	77.8	22.3	72.7	27.3
	77.7	22.4	74.4	25.6	72.3	27.7
**Average:**	**77.3**	**22.7**	**74.1**	**25.9**	**72.9**	**27.1**

Table S2. Platinum load (wt%) and encapsulation efficiency (%) in SBA-15|**1**, SBA-15|**2** and SBA-15|**3**.

	SBA-15|**1**	SBA-15|**2**	SBA-15|**3**
Theoretical Pt/Si ratio	0.42	0.42	0.42
Calculated Pt/Si ratio	0.29	0.35	0.37
Theoretical Pt load (wt%)	10.67	11.01	11.57
Calculated Pt load (wt%)	7.51	9.23	10.33
Encapsulation efficiency (%)	70.36	83.83	89.24

Drug release kinetics

To study the kinetics of the drug release, *in vitro* drug release data was quantitatively correlated with four mathematical models viz. zero-order, first-order, Higuchi and Korsmeyer–Peppas models. The obtained constants and degrees of correlation R^2 for each model are given in Table S3.

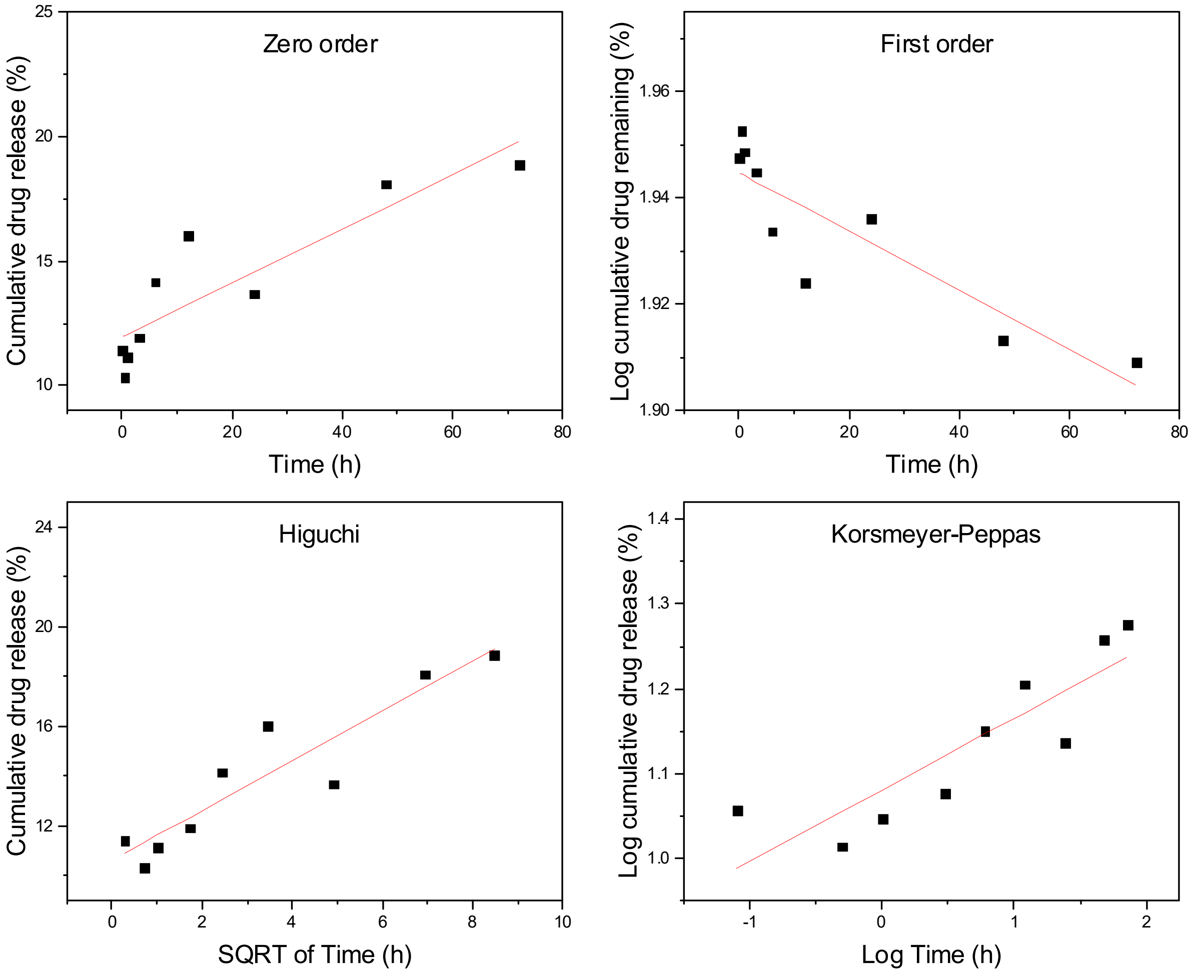

Figure S24. Zero order, first order, Higuchi and Korsmeyer–Peppas kinetic release of **1** from SBA-15|**1**.

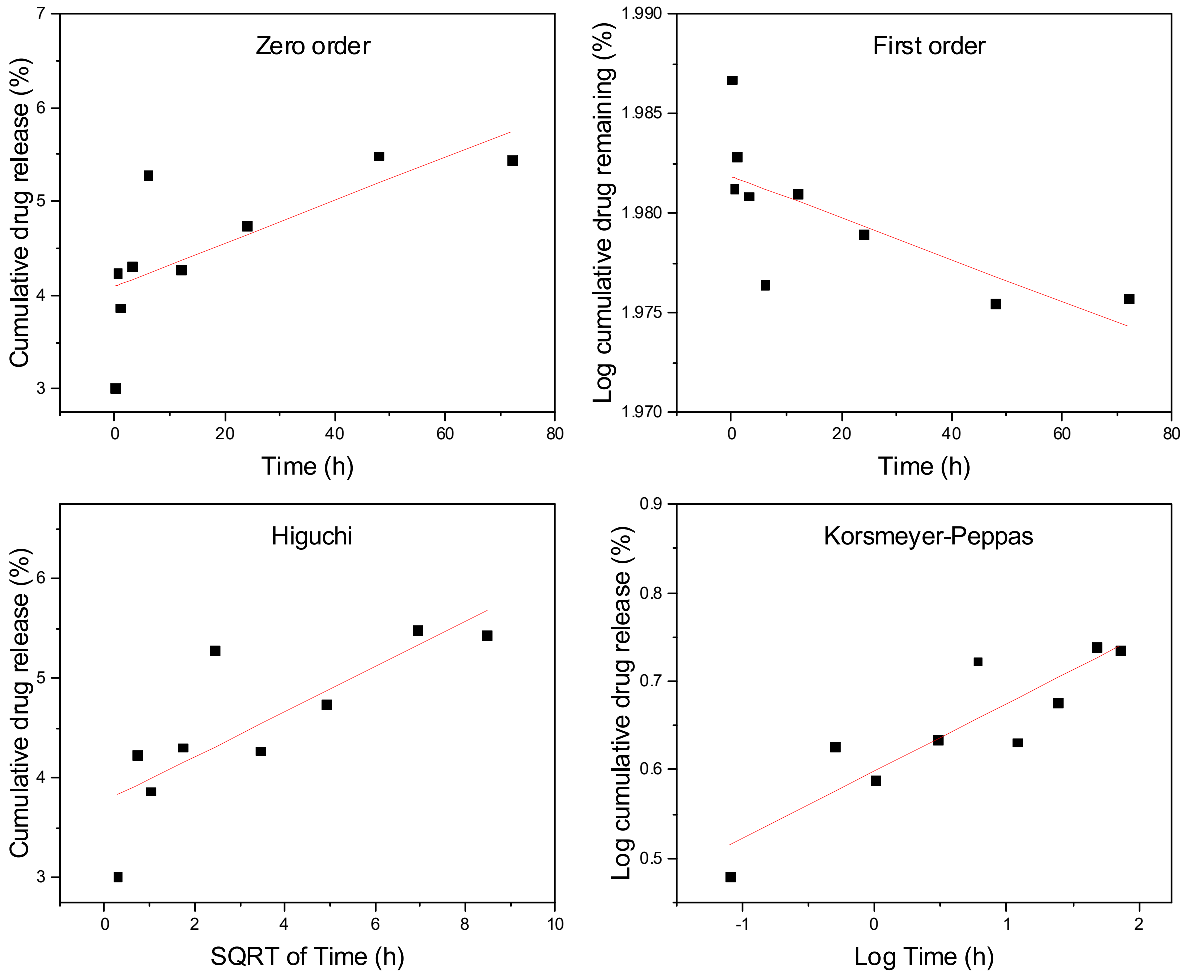

Figure S25. Zero order, first order, Higuchi and Korsmeyer–Peppas kinetic release of **2** from SBA-15|**2**.

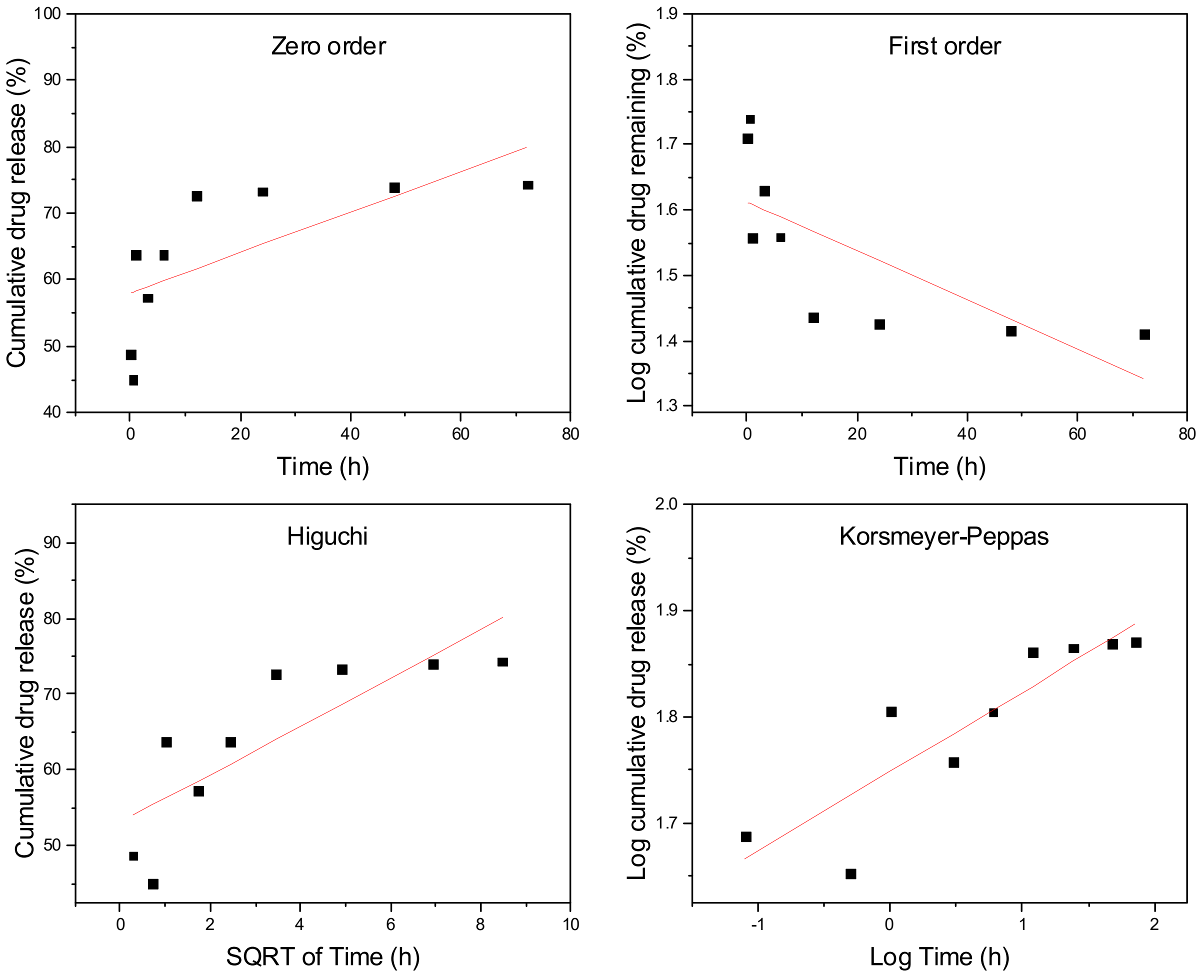

Figure S26. Zero order, first order, Higuchi and Korsmeyer–Peppas kinetic release of **3** from SBA-15|**3**.

Table S3. Constants and coefficient of determinations (R^2) for each model.

	Zero order		First Order		Higuchi		Korsmeyer–Peppas	
	K^0	R^2	Q_{inf}	R^2	K_h	R^2	n	R^2
SBA-15|**1**	0.109	0.785	0.001	0.793	1.003	0.867	0.085	0.763
SBA-15|**2**	0.023	0.514	0.001	0.516	0.225	0.641	0.075	0.790
SBA-15|**3**	0.304	0.477	0.009	0.539	3.173	0.672	0.074	0.793

Cell viability

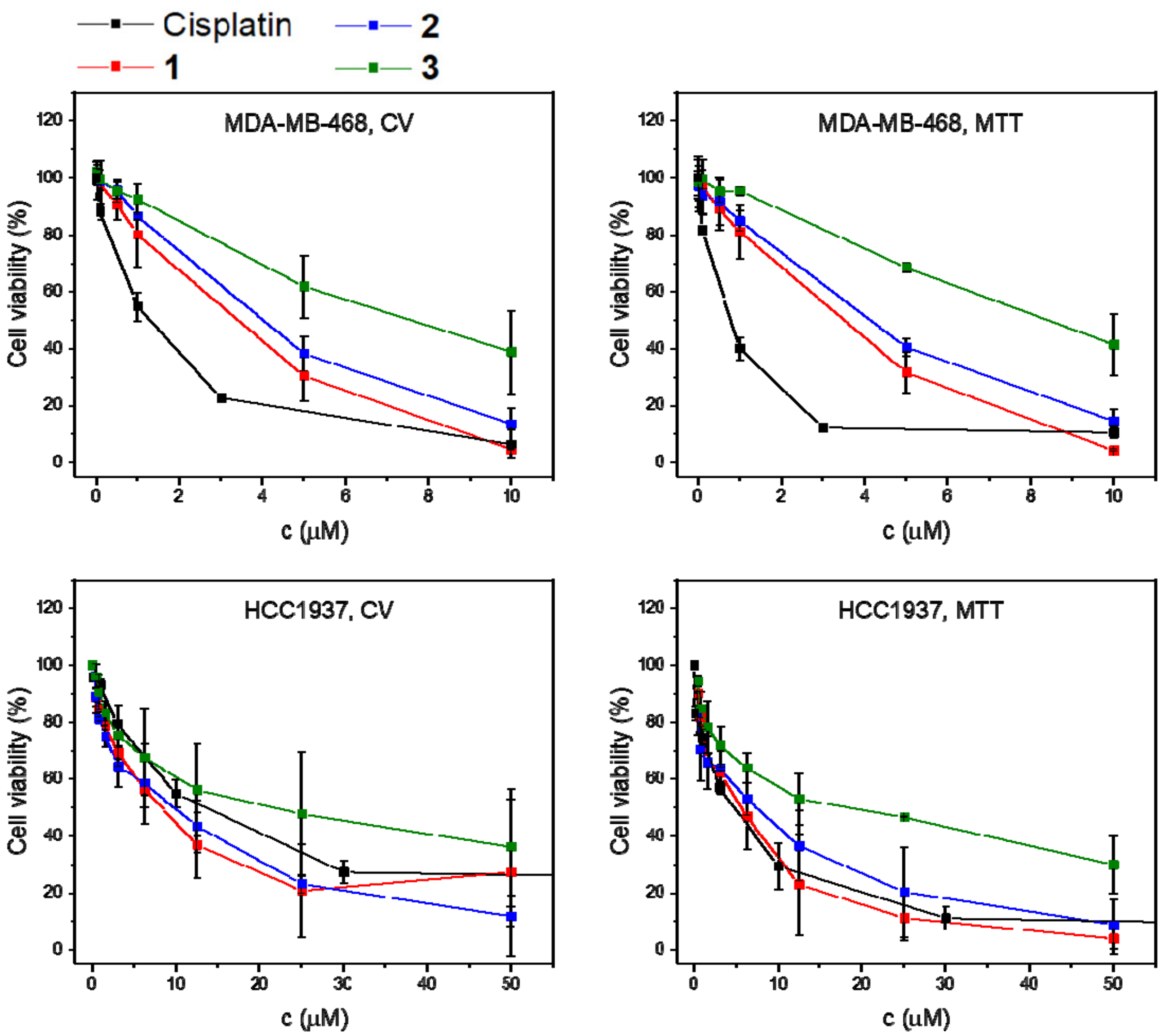

Figure S27. Cell viability of cisplatin, **1**, **2** and **3** determined by CV and MTT assays in MDA-MB-468 and HCC1937 human breast cancer cell lines.

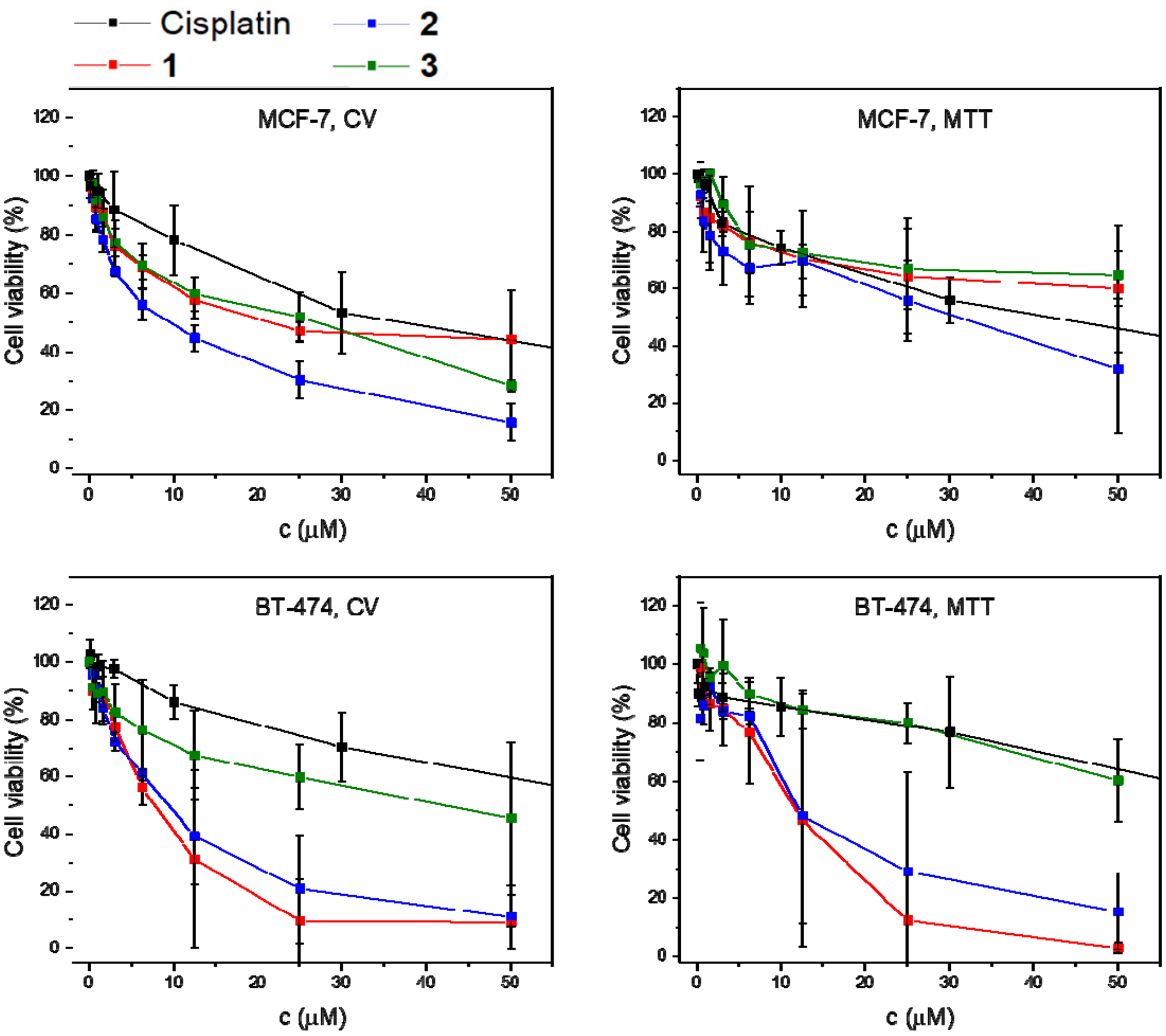

Figure S28. Cell viability of cisplatin, **1**, **2** and **3** determined by CV and MTT assays in MCF-7 and BT-474 human breast cancer cell lines.

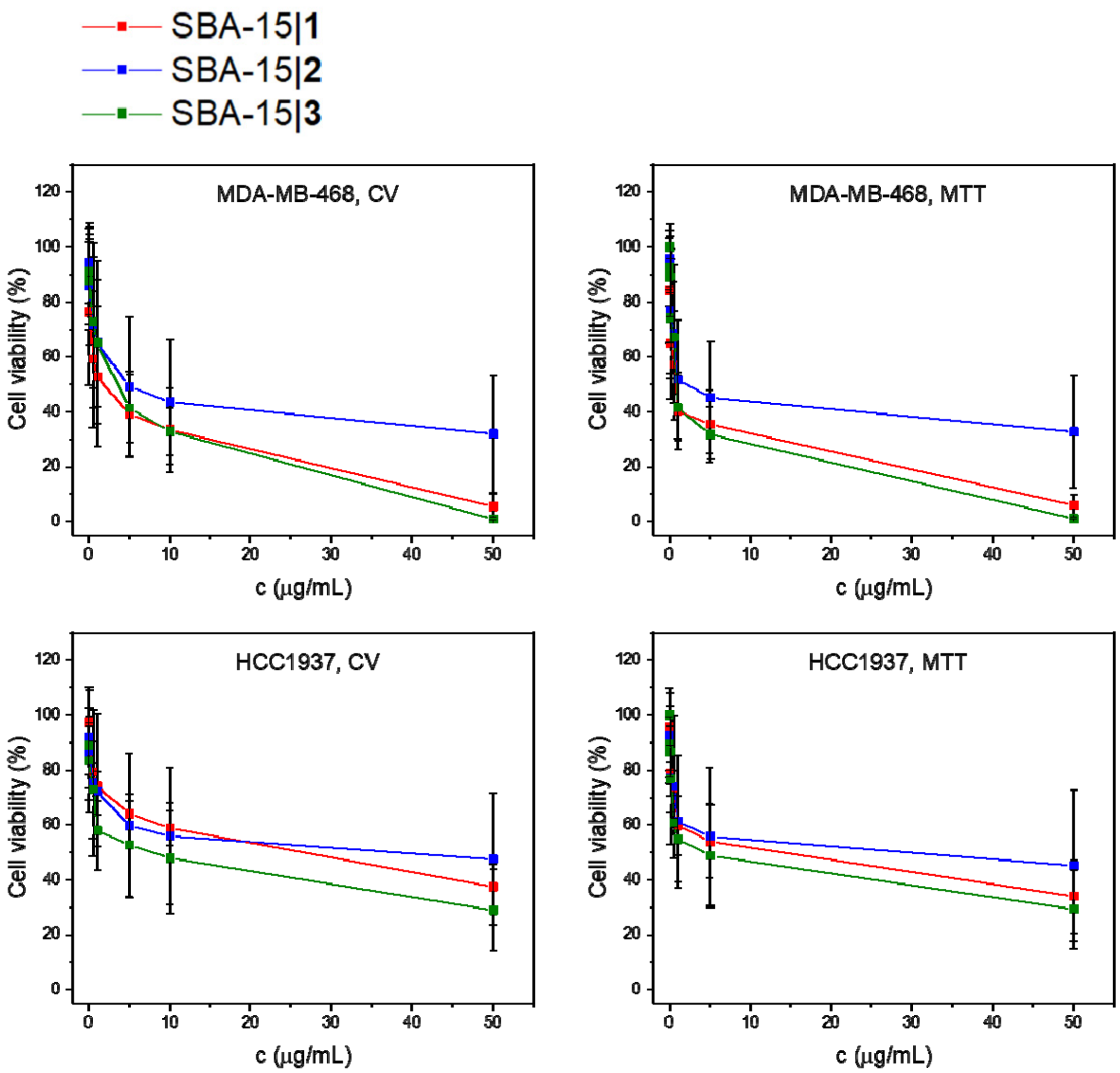

Figure S29. Cell viability of SBA-15|**1**, SBA-15|**2** and SBA-15|**3** determined by CV and MTT assays in MDA-MB-468 and HCC1937 human breast cancer cell lines.

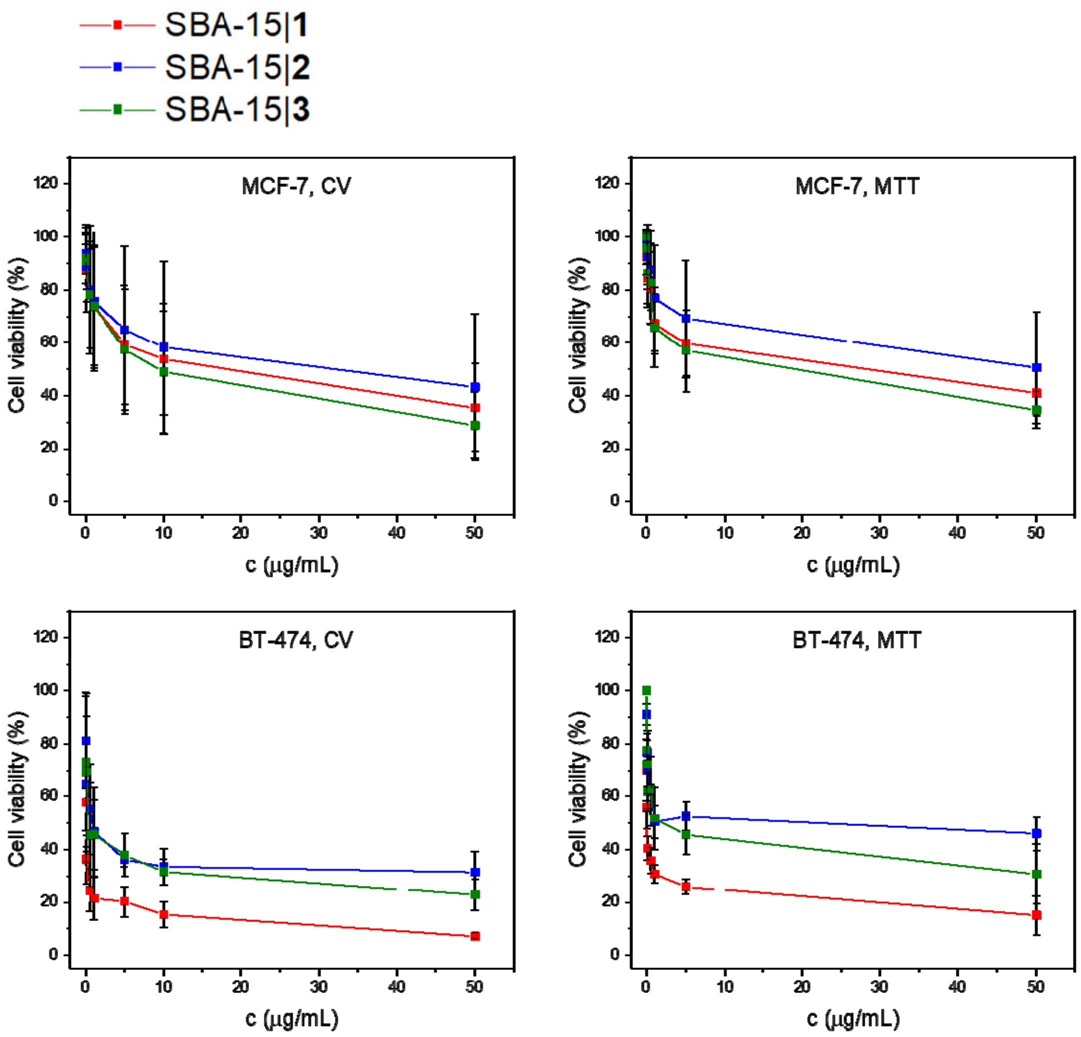

Figure S30. Cell viability of SBA-15|**1**, SBA-15|**2** and SBA-15|**3** determined by CV and MTT assays in MCF-7 and BT-474 human breast cancer cell lines.

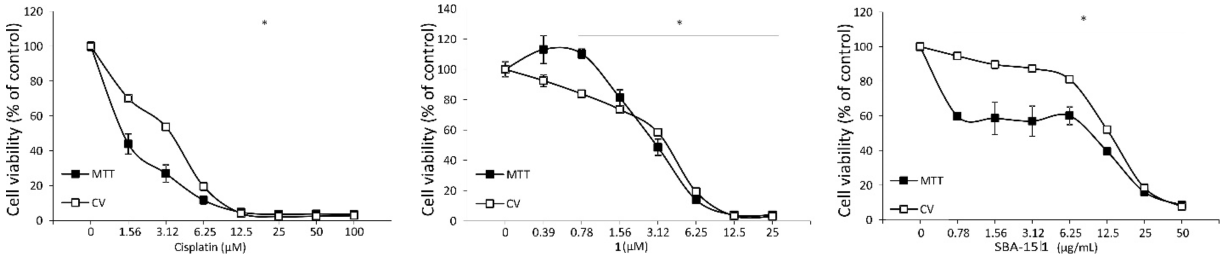

Figure S31. Cell viability of cisplatin, **1** and SBA-15|**1** determined by CV and MTT assays in mouse-derived 4T1 breast cancer cell line.

Urine parameters upon *in vivo* treatment

Table S4. Urine parameters of untreated animals (control) and animals exposed to treatment with cisplatin, **1** and SBA-15|**1**.

	Control	Cisplatin	**1**	SBA-15|**1**
Leukocytes (leu/µL)	25 ± 0	25 ± 0	25 ± 0	25 ± 0
Nitrites	0 ± 0	0 ± 0	0 ± 0	0 ± 0
Urobilinogen (mg/dL)	4 ± 0	2.5 ± 2.1	6 ± 2.8	2.5 ± 2.1
Proteins (mg/dL)	100 ± 0	10 ± 0	30 ± 0	65 ± 49.5
pH	6 ± 0	6 ± 0	6 ± 0	7 ± 0
Blood (ery/µL)	0 ± 0	0 ± 0	0 ± 0	0 ± 0
Specific Gravity	1030 ± 0	1030 ± 0	1030 ± 0	1030 ± 0
Ketone (mg/dL)	30 ± 28.3	10 ± 0	7.5 ± 3.5	10 ± 0
Bilirubin (mg/dL)	0.8 ± 0.4	1 ± 0	0.8 ± 0.4	1 ± 0
